# A live-attenuated SARS-CoV-2 vaccine candidate with accessory protein deletions

**DOI:** 10.1038/s41467-022-31930-z

**Published:** 2022-07-27

**Authors:** Yang Liu, Xianwen Zhang, Jianying Liu, Hongjie Xia, Jing Zou, Antonio E. Muruato, Sivakumar Periasamy, Chaitanya Kurhade, Jessica A. Plante, Nathen E. Bopp, Birte Kalveram, Alexander Bukreyev, Ping Ren, Tian Wang, Vineet D. Menachery, Kenneth S. Plante, Xuping Xie, Scott C. Weaver, Pei-Yong Shi

**Affiliations:** 1 Department of Biochemistry and Molecular Biology, University of Texas Medical Branch, Galveston, TX USA; 2 Department of Microbiology and Immunology, University of Texas Medical Branch, Galveston, TX USA; 3 Institute for Human Infections and Immunity, University of Texas Medical Branch, Galveston, TX USA; 4 Department of Pathology, University of Texas Medical Branch, Galveston, TX USA; 5 Galveston National Laboratory, Galveston, TX USA; 6 World Reference Center for Emerging Viruses and Arboviruses, University of Texas Medical Branch, Galveston, TX USA; 7 Sealy Institute for Vaccine Sciences, University of Texas Medical Branch, Galveston, TX USA; 8 Sealy Institute for Drug Discovery, University of Texas Medical Branch, Galveston, TX USA; 9 Sealy Center for Structural Biology & Molecular Biophysics, University of Texas Medical Branch, Galveston, TX USA

**Keywords:** Live attenuated vaccines, SARS-CoV-2

## Abstract

We report a live-attenuated SARS-CoV-2 vaccine candidate with (i) re-engineered viral transcription regulator sequences and (ii) deleted open-reading-frames (ORF) 3, 6, 7, and 8 (∆3678). The ∆3678 virus replicates about 7,500-fold lower than wild-type SARS-CoV-2 on primary human airway cultures, but restores its replication on interferon-deficient Vero-E6 cells that are approved for vaccine production. The ∆3678 virus is highly attenuated in both hamster and K18-hACE2 mouse models. A single-dose immunization of the ∆3678 virus protects hamsters from wild-type virus challenge and transmission. Among the deleted ORFs in the ∆3678 virus, ORF3a accounts for the most attenuation through antagonizing STAT1 phosphorylation during type-I interferon signaling. We also developed an mNeonGreen reporter ∆3678 virus for high-throughput neutralization and antiviral testing. Altogether, the results suggest that ∆3678 SARS-CoV-2 may serve as a live-attenuated vaccine candidate and a research tool for potential biosafety level-2 use.

## Abstract (web summary)

A live-attenuated COVID vaccine could enrich the current vaccine portfolio. Here, Liu et al. engineer a live-attenuated SARS-CoV-2 vaccine candidate by deleting four viral accessory genes and show immunogenicity and protection in mice and hamsters.

## Introduction

The pandemic of COVID-19, caused by SARS-CoV-2, has led to over 513 million confirmed infections and 6.2 million deaths (as of 1 May 2022; https://coronavirus.jhu.edu/). Different vaccine platforms have been successfully developed for COVID-19, including mRNA, viral vector, subunit protein, and inactivated virus. Live-attenuated vaccines of SARS-CoV-2 have not been actively explored, even though they may have advantages of low cost, strong immunogenicity, and potentially long immune durability^1^. The SARS-CoV-2 virion consists of an internal nucleocapsid, formed by the genomic RNA coated with nucleocapsid (N) proteins, and an external envelope, formed by a cell-derived bilipid membrane embedded with spike (S), membrane (M), and envelope (E) proteins^2^. The plus-sense, single-stranded viral RNA genome encodes open-reading-frames (ORFs) for sixteen nonstructural proteins that form the replication machinery (ORF1a/ORF1b), four structural proteins (S, E, M, and N), and seven accessory proteins^3^. Although the exact functions of SARS-CoV-2 accessory proteins remain to be determined, previous studies of other coronaviruses suggest that these proteins are not essential for viral replication but can modulate replication and pathogenesis through interacting with host pathways^4–8^. Thus, deletion of the accessory proteins could be used to attenuate SARS-CoV-2.

**Fig. 1:**
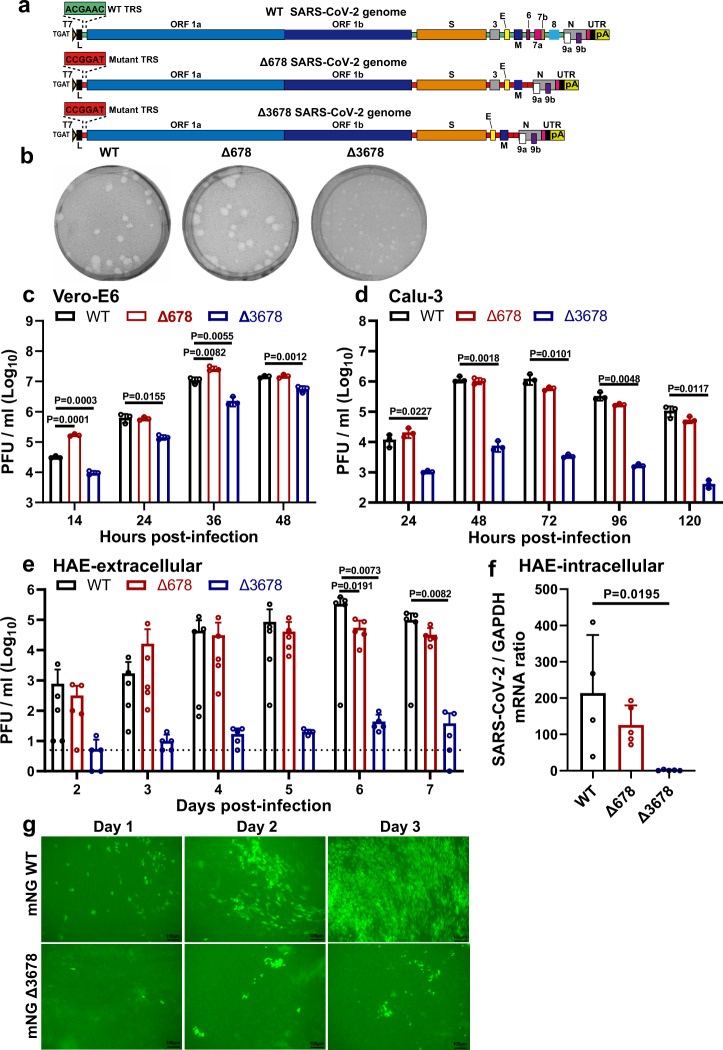
Attenuation of Δ3678 SARS-CoV-2 in cell culture. **a** Scheme diagram for the construction of Δ678 and Δ3678 SARS-CoV-2. The deletions were introduced to the backbone of USA-WA1/2020 strain. Green and red boxes indicate the original and mutated TRS sequences, respectively. T7 T7 promoter, L leader sequence, TRS transcription regulatory sequences; ORF open-reading frame, E envelope glycoprotein gene, M membrane glycoprotein gene, N nucleocapsid gene, UTR untranslated region, pA poly A tails. **b** Plaque morphologies of recombinant WT, Δ678, and Δ3678 viruses. Plaque assays were performed on Vero-E6 cells and stained on day 2.5 post-infection. **c**–**e** Replication kinetics of WT, Δ678, and Δ3678 SARS-CoV-2s on Vero-E6 (**c**), Calu-3 (**d**), and HAE (**e***)* cells. WT, Δ678, and Δ3678 viruses were inoculated onto Vero-E6, Calu-3, and HAE cells at MOIs of 0.01, 0.1, and 2, respectively. After a 2-h incubation, the cells were washed three times with DPBS and continuously cultured under fresh 2% FBS DMEM. Culture supernatants were measured for infectious virus titers using plaque assays on Vero-E6 cells. **f** Intracellular levels of WT, Δ678, and Δ3678 RNA in HAE cells on day 7 post-infection. The HAE cells were washed with PDBS for three times, lysed with Trizol for RNA isolation, quantified for viral RNAs using RT-qPCR. **c**–**f** Dots represent individual biological replicates (*n* = 3 for Vero-E6 and Calu-3; *n* = 5 for HAE). The values in the graph represent the mean ± standard deviation. An unpaired two-tailed *t* test was used to determine significant differences between WT and Δ678/Δ3678 groups. *P* values were adjusted using the Bonferroni correction to account for multiple comparisons. Differences were considered significant if *p* < 0.025. **g** mNG-positive HAE cells after infection with mNG WT or mNG Δ3678 virus at an MOI of 0.5. Representative images of mNG-positive cells from three independent experiments are shown; the low resolution of the mNG-positive images was because the HAE culture consisted of multilayer live cells on a plastic insert, which was placed in 6-well plates. Scale bar, 100 µm. **c**–**f** Source Data are provided as a Source Data file.

Coronaviruses replicate and transcribe subgenomic RNAs (sgRNAs) via a discontinuous transcription mechanism that is mediated by transcription regulatory sequences (TRSs). TRSs are conserved nucleotide sequences that are located near the 5′-end of the genome and at the 5′-ends of downstream ORFs (Fig. 1a). These TRSs regulate transcription via base-pairing between the leader TRS and body TRSs that leads to the production of sgRNAs, which translate downstream ORFs^9–11^. Within the TRS, a core sequence of 6 to 8 nucleotides guides the base-pair formation between nascent RNA and the leader TRS. It was previously shown that rewiring the guide sequence of the SARS-CoV TRS network could produce an attenuated virus. Such TRS-mutant SARS-CoV could serve as a live-attenuated vaccine approach to minimize the risk of reversion^12,13^.

Reverse genetic systems are important tools to engineer and study viruses. In response to the COVID-19 pandemic, three types of reverse genetic systems have been developed for SARS-CoV-2: (i) an infectious cDNA clone^14–18^, (ii) a transient replicon (a self-replicating viral RNA with one or more structural genes deleted)^19,20^, and (iii) a *trans*-complementation system (replicon RNAs in cells that express the missing genes in the replicon, allowing for single cycle replication without spread)^21–23^. The three systems have their own strengths and weaknesses and are complementary to each other when applied to address different research questions. The infectious cDNA clone requires biosafety level-3 (BSL-3) containment to recover and handle infectious SARS-CoV-2. The transient replicon system requires RNA preparation and transfection for each experiment; cell lines harboring replicons that can be continuously cultured, like those developed for hepatitis C virus and other plus-sense RNA viruses^24–26^, have yet to be established for SARS-CoV-2. The *trans*-complementation system produces virions that can infect naïve cells for only a single round. Compared with the infectious cDNA clone, both the replicon and *trans*-complementation system have the advantage of allowing experiments to be performed at biosafety level-2 (BSL-2). A new system that combines the strengths of the current three systems (e.g., multiple rounds of viral infection of naïve cells that can be performed at BSL-2) would be very useful for COVID-19 research and countermeasure development. Here we report a highly attenuated SARS-CoV-2 (with deleted accessory proteins and rewired TRS) that can potentially serve as a live-attenuated vaccine platform and a BSL-2 experimental system.

## Results

### Attenuation of SARS-CoV-2 by deletion of accessory genes

Using an infectious clone of USA-WA1/2020 SARS-CoV-2, we constructed two mutant viruses containing accessory ORF deletions (Fig. 1a), one with ORF 6, 7, and 8 deletions (∆678) and another with ORF 3, 6, 7, and 8 deletions (∆3678). Besides the ORF deletions, the TRS of both ∆678 and ∆3678 viruses were mutated from the wild-type (WT) ACGAAC to CCGGAT (mutant nucleotides underlined; Fig. 1a). The mutated TRS virtually eliminates the possibility to produce hybrid live virus through recombination between the ∆678 or ∆3678 RNA and WT SARS-CoV-2 in case of co-infection in natural setting when the mutant virus is used as a potential live-attenuated vaccine candidate^12,13^. We chose to mutate 3 nucleotides (TRS3), rather than seven nucleotides (TRS7) in the TRS sequence, because (i) previous studies showed that TRS7 significantly attenuated SARS-CoV^12,13^ and (ii) this study was not aimed to attenuate SARS-CoV-2 via the TRS mutation approach. On Vero-E6 cells, the ∆678 virus developed plaques similar to the WT virus, whereas the ∆3678 virus produced smaller plaques (Fig. 1b). Replication kinetics analysis showed that WT and ∆678 replicated to comparable viral titers on Vero-E6 (Fig. 1c), Calu-3 (Fig. 1d), and primary human airway epithelial (HAE) cultures (Fig. 1e). In contrast, the replication of ∆3678 was slightly attenuated on Vero-E6 cells (Fig. 1c), but became more attenuated on Calu-3 (360-fold lower peak viral titer than the WT virus at 72 h; Fig. 1d) and HAE cultures (7,500-fold lower peak viral titer than the WT virus on day 6; Fig. 1e). Consistently, the intracellular level of ∆3678 RNA was about 100-fold lower than those of ∆678 and WT RNA in HAE cells (Fig. 1f). Corroboratively, the ∆3678 virus caused much less cytopathic effects (CPE) than the ∆678 and WT viruses on both Vero-E6 and Calu-3 cells (Supplementary Fig. 1). To further confirm the attenuation of ∆3678 virus, we engineered the mNeonGreen (mNG) gene into the ∆3678 and WT viruses^27^. When infecting HAE cultures, the mNG ∆3678 virus developed significantly fewer mNG-positive cells than the mNG WT virus (Fig. 1g). Taken together, the results indicate that (i) deletions of ORFs 6, 7, and 8 do not significantly attenuate SARS-CoV-2 in cell culture; (ii) an additional deletion of ORF3 to the ∆678 virus significantly increases the attenuation of ∆3678; and (iii) the ∆3678 virus is strikingly more attenuated when infecting immune-competent cells than when infecting interferon-deficient cells. The cell culture results prompted us to evaluate the attenuation and vaccine potential of ∆3678 virus in animal models.

### Characterization of ∆3678 SARS-CoV-2 as a potential live-attenuated vaccine in a hamster model

**Fig. 2:**
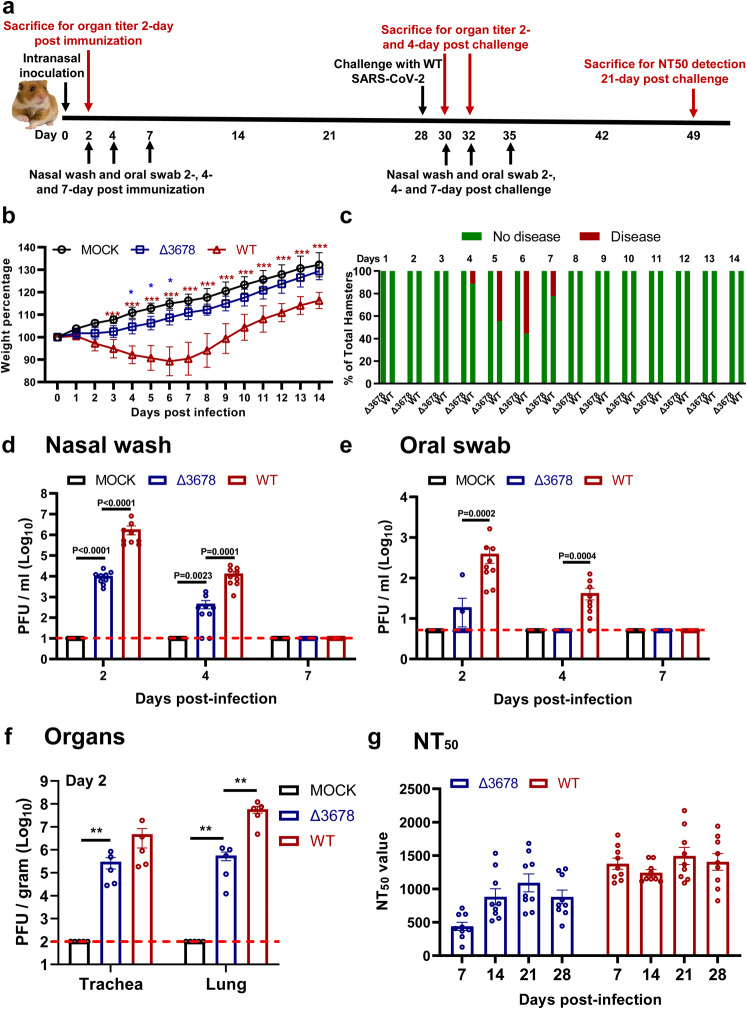
Attenuation of Δ3678 SARS-CoV-2 in hamsters. **a** Experimental scheme of Δ3678 virus immunization and WT virus challenge. Hamsters were intranasally inoculated with 10^6^ PFU of WT or Δ3678 virus. On day 2 post-inoculation, organ viral loads (*n* = 5) were measured by plaque assays. Nasal washes and oral swabs (*n* = 10) were collected on days 2, 4, and 7 post-inoculation. On day 28 post-immunization, the hamsters were challenged by 10^5^ PFU of WT SARS-CoV-2. On days 2 and 4 post-challenge, plaque assays were performed to measure organ viral loads (*n* = 5). On day 21 post-challenge, the animals were terminated to measure neutralization titer (NT_50_). **b** Weight changes of hamsters after intranasal infection with WT (*n* = 9) or Δ3678 (*n* = 9) SARS-CoV-2. Uninfected mock group (*n* = 9) was included as a negative control. Body weights were measured daily for 14 days. The data are shown as mean ± standard deviation. The weight changes between Δ3678 and mock or WT groups were analyzed using two-factor analysis of variance (ANOVA) with Tukey’s post hoc test. The blue and red asterisks stand for the statistic difference between Δ3678 and mock or WT, respectively. The exact *P* values of the blue asterisks are 0.039 (day 4), 0.0178 (day 5), and 0.0353 (day 6), respectively; the exact *P* values of the red asterisks are *P* < 0.0001.**c**, Disease of Δ678 and Δ3678 virus-infected animals. The diseases include ruffled fur, lethargic, hunched posture, and orbital tightening. The percentages of animals with or without diseases are presented. **d**–**f** Viral loads in nasal wash (**d**), oral swab (**e**), trachea, and lung (**f**) after infection with Δ3678 or WT virus. Dots represent individual animals (*n* = 5). The mean ± standard error is presented. A non-parametric two-tailed Mann–Whitney test was used to determine the differences between mock, Δ3678, or WT groups. *P* values were adjusted using the Bonferroni correction to account for multiple comparisons. Differences were considered significant if p < 0.025. **g**, Neutralization titers of sera from WT- and Δ3678 virus-inoculated hamsters on days 7, 14, 21, and 28 post-inoculation. The neutralization titers were measured against WT SARS-CoV-2. **b**–**g** Source Data are provided as a Source Data file.

We characterized the attenuation of ∆3678 virus in a hamster model (Fig. 2a). After intranasal infection with 10^6^ plaque-forming units (PFU) of ∆3678, the hamsters did not lose weight (Fig. 2b) or develop disease (Fig. 2c). In contrast, the WT virus-infected animals lost weight (Fig. 2b) and developed disease (Fig. 2c), as observed in our previous studies^28–30^. On day 2 post-infection, viral loads in the ∆3678-infected nasal washes (Fig. 2d), oral swabs (Fig. 2e), tracheae, and lungs (Fig. 2f) were 180-, 20-, 16-, and 100-fold lower than those in the WT-infected specimens. The ∆3678 infection elicited robust neutralization with a peak 50% neutralization titer (NT_50_) of 1090 on day 21 post-infection, while the WT virus evoked 1.4-fold higher peak NT_50_ (Fig. 2g). The results demonstrate that the ∆3678 virus is attenuated and can elicit robust neutralization in hamsters.

**Fig. 3:**
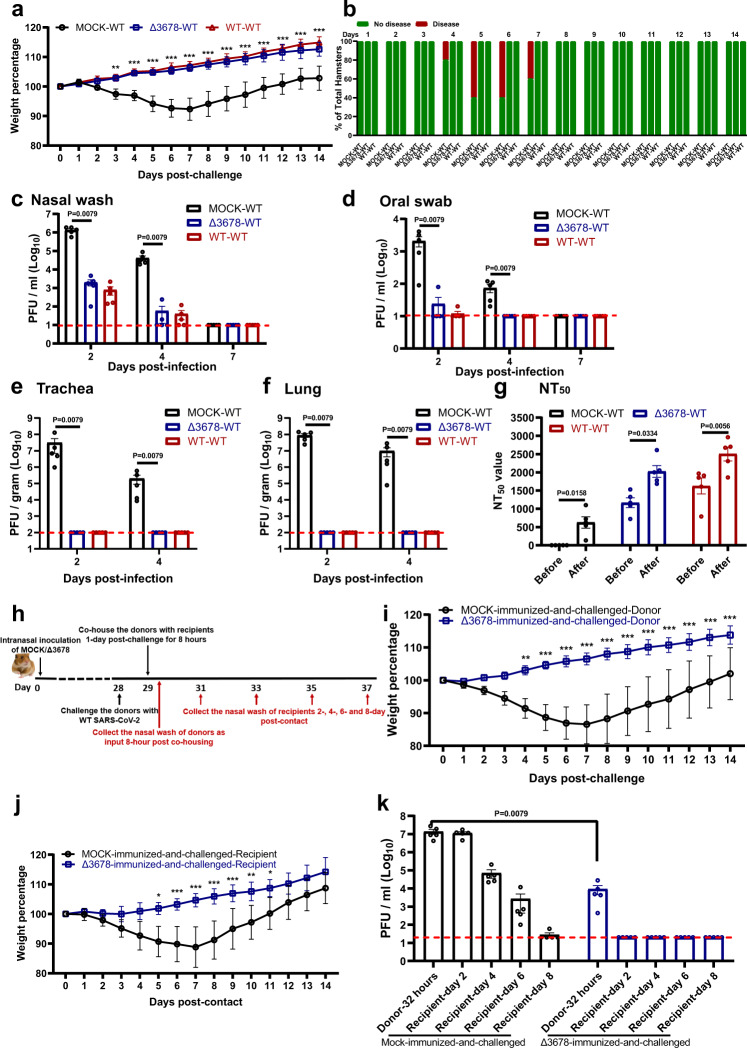
Protection of Δ3678 virus-immunized hamsters from WT SARS-CoV-2 challenge and transmission. **a** Weight loss of immunized and-challenged hamsters (*n* = 5). The data are shown as mean ± standard deviation. The weight changes were statistically analyzed using two-way ANOVA (two-sided) with Tukey’s post hoc test. The statistic difference between the Δ3678- and mock-immunized groups are indicated. The exact *P* values are 0.0049 (day 3) and *P* < 0.0001 (days 4–14). **b** The percentages of animals with or without diseases were presented. **c**–**f** Viral loads in the nasal wash (**c**), oral swab (**d**), trachea (**e**), and lung (**f**) after challenge. Dots represent individual animals (*n* = 5). The values of mean ± SEM are presented. A non-parametric two-tailed Mann–Whitney test was used to determine the statistical differences. *P* values were adjusted using the Bonferroni correction to account for multiple comparisons. Differences were considered significant if *p* < 0.025. **g** Neutralization titers of immunized hamsters before and after challenge. Dots represent individual animals (*n* = 5). The values in the graph represent the mean ± SEM. A two-sided paired *t* test was used to analyze the difference. **h** Experimental design of transmission blockage in hamsters. Hamsters were immunized with 10^6^ PFU of Δ3678 virus (*n* = 5) or medium mock (*n* = 5). On day 28, the hamsters were challenged with 10^5^ PFU of WT and co-housed with recipient hamsters 1 day after the challenge. The nasal washes were collected at indicated time points. **i**, **j** Weight loss of donors post-challenge (**i**) and recipients post-contact (**j**). The data are shown as mean ± standard deviation. The weight changes were statistically analyzed using two-way ANOVA (two-sided) with Tukey’s post hoc test. **i** The exact *P* values of are 0.0012 (day 5), *P* < 0.0001 (days 6–13), 0.0009 (day 14). **j** The exact *P* values of are 0.0263 (day 4), 0.0005 (day 5), *P* < 0.0001 (days 6–8), 0.0001(day 9), 0.0015 (day 10), 0.0197 (day 11). **k** Viral loads in a nasal wash of donors post-challenge and recipients post-contact. Dots represent individual animals (*n* = 5). The data are shown as mean ± SEM. A non-parametric two-tailed Mann–Whitney test was used to analyze the difference. **a**–**k** Source Data are provided as a Source Data file.

We examined whether the above-immunized hamsters could be protected from SARS-CoV-2 challenge. After intranasal challenge with 10^5^ PFU of WT SARS-CoV-2 on day 28 post-immunization (Fig. 2a), both the ∆3678- and WT virus-immunized animals were protected from weight loss (Fig. 3a) or disease (Fig. 3b). Compared with the mock-immunized group, the viral loads in the nasal washes (Fig. 3c) and oral swabs (Fig. 3d) from the ∆3678- and WT virus-immunized groups were decreased by >660 (day 2) and >80 folds (day 2), respectively; no infectious viruses were detected in trachea (Fig. 3e) and lungs (Fig. 3f) from the immunized groups. The challenge significantly increased the neutralization titers (on day 21 post-challenge) in both the ∆3678- and WT virus-immunized groups (Fig. 3g), suggesting that a single infection with the ∆3678 or WT virus did not elicit sterilizing immunity. Histopathology analysis showed that immunization with attenuated ∆3678 virus reduced lung pathology score, inflammation, alveolar septa change, and airway damage (Supplementary Fig. 2). In contrast, previous infection with WT virus did not exhibit improved lung histopathology after the challenge, possibly because the observed pathologic changes were caused by the initial WT viral infection (Supplementary Fig. 2). Collectively, the results demonstrate that immunization with attenuated ∆3678 virus can protect against WT SARS-CoV-2 challenge in hamsters.

Next, we tested whether lower dose immunization could also achieve protection. Hamsters were immunized with 10^2^, 10^3^, 10^4^, or 10^5^ PFU of ∆3678 viruses. No weight loss was observed for all dose groups (Supplementary Fig. 3a). All dose groups developed equivalent, low lung viral loads (Supplementary Fig. 3b). After challenging with WT SARS-CoV-2, all dose groups exhibited protection similar to the 10^6^-PFU-dose group, including undetectable viral loads in the tracheae or lungs and significantly reduced viral loads in the nasal washes and oral swabs (Supplementary Fig. 3c). These results indicate a low dose of 10^2^ PFU of ∆3678 immunization is protective in hamsters.

Since infectious viruses were detected in the nasal and oral specimens from the ∆3678-immunized hamsters after the challenge, we examined whether such low levels of the virus could be transmitted to naive hamsters. On day 1 post challenge, the ∆3678-immunized-and-challenged animals (donor) were co-housed with clean naive hamsters (recipient) for 8 h, after which the donor and recipient animals were separated (Fig. 3h). As expected, after WT virus challenge, no weight loss was observed in the ∆3678-immunized donor animals, but weight loss did occur in the mock-immunized donor animals (Fig. 3i). After co-housing with ∆3678-immunized-and-challenged donor animals, naïve recipient animals did not lose weight (Fig. 3j) and did not have infectious viruses in the nasal washes (Fig. 3k). In contrast, after co-housing with the mock-immunized-and-challenged donor animals, the recipient animals lost weight (Fig. 3j) and developed high viral loads in the nasal wash (Fig. 3k). Altogether, the results indicate that although the ∆3678-immunized donor animals developed low viral loads in their nasal and oral specimens after the WT virus challenge, they were unable to transmit the virus to naïve hamsters.

### Attenuation of ∆3678 SARS-CoV-2 in K18-hACE2 mice

**Fig. 4:**
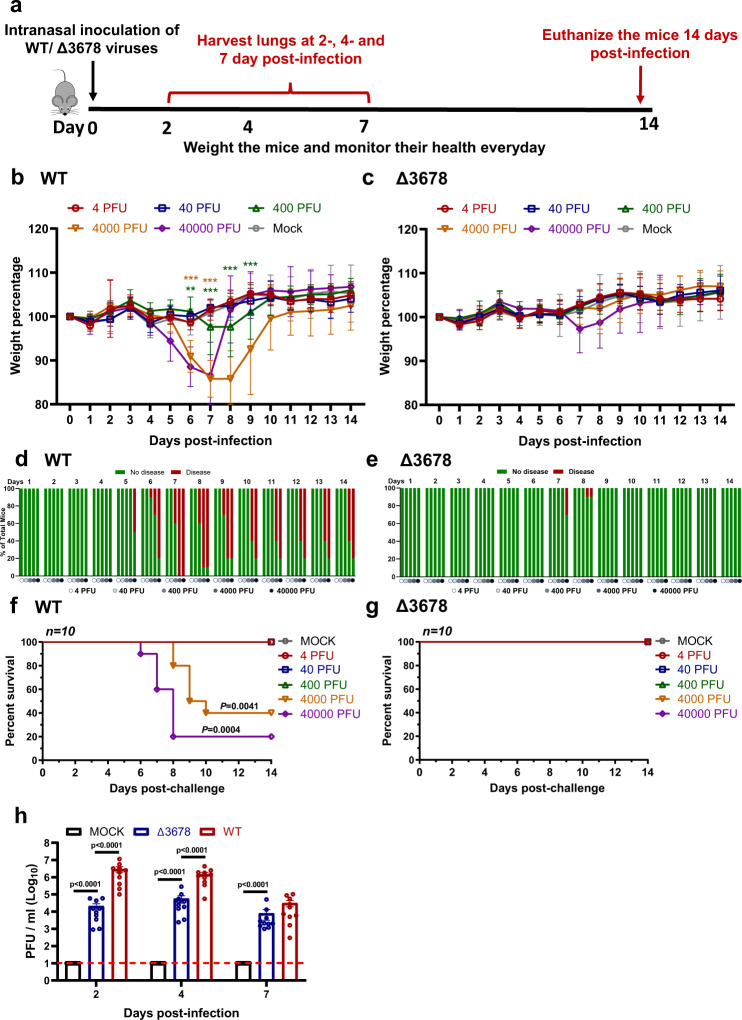
Attenuation of Δ3678 SARS-CoV-2 in K18-hACE2 mice. **a** Experimental scheme. K18-hACE2 mice were intranasally inoculated with 4, 40, 400, 4000, or 40000 PFU of WT (*n* = 10) or Δ3678 virus (*n* = 10). Lung viral loads were measured on days 2, 4, and 7 post-infection. The infected mice were monitored for bodyweight (**b**, **c**), disease (**d**, **e**), and survivals (**f**, **g**) for 14 days. **b**, **c** Bodyweight changes. Different viral infection doses are indicated by different colors. The data are shown as mean ± standard deviation. The weight changes between mock- and virus-infected groups were statistically analyzed using a two-factor analysis of variance (ANOVA, two-sided) with Tukey’s post hoc test. The orange and purple asterisks indicate the statistical difference between mock and 4000- or 40000 PFU infection groups. The exact *P* values of the orange asterisks are 0.0070 (day 6), *P* < 0.001 (days 7–9), the exact *P* values of the purple asterisks are *P* < 0.001 (days 6, 7). **d**, **e** Disease of WT and Δ3678 virus-infected animals. The diseases include ruffled fur, lethargic, hunched posture, or orbital tightening. The percentages of hamsters with or without diseases were presented. **f**, **g** Survival. A mixed-model ANOVA using Dunnett’s test for multiple comparisons was used to evaluate the statistical significance. **h** Lung viral loads from 40000 PFU WT- and Δ3678K-infected K18-hACE2 mice. Dots represent individual animals (*n* = 10). The mean ± standard error of the mean is presented. A non-parametric two-sided Mann–Whitney test was used to determine statistical significance. *P* values were adjusted using the Bonferroni correction to account for multiple comparisons. Differences were considered significant if *p* < 0.025. **b**–**h** Source Data are provided as a Source Data file.

To further characterize the attenuation of ∆3678, we intranasally inoculated K18-hACE2 mice with 4, 40, 400, 4000, or 40,000 PFU of WT or ∆3678 virus (Fig. 4a). The infected groups were compared for their weight loss, survival rates, and disease signs. The WT viral infection caused weight loss at doses of ≥400 PFU (Fig. 4b), diseases at dose ≥400 PFU (Fig. 4d), and deaths at doses ≥4000 PFU (Fig. 4f). In contrast, the ∆3678 virus caused slight (statistically insignificant) weight loss at 40,000 PFU (Fig. 4c), transient disease at ≥4000 PFU (Fig. 4e), and no death at any dose (Fig. 4g). Consistently, the lung viral loads from the 40,000 PFU ∆3678-infected mice were significantly lower than those from the WT-infected animals (Fig. 4h); compared with the WT virus-infected mice, the ∆3678 virus-infected animals developed significantly reduced histopathology, pathology score, inflammation, alveolar septa change, and airway damage (Supplementary Fig. 4). The results demonstrated that ∆3678 virus was highly attenuated in the K18-hACE2 mice.

### Transmissibility analysis of ∆3678 SARS-CoV-2 in hamsters

To test the transmissibility of ∆3678 virus, we infected donor hamsters with 10^6^ PFU of Δ3678 virus. On day 1 post-infection, the donor hamsters were co-housed with naïve recipient hamsters for 8 h, after which the donor animals were removed (Supplementary Fig. 5a). The recipient animals were measured for ∆3678 viral loads in nasal washes. Compared with WT virus, the ∆3678 viral loads in the nasal washes from recipient hamsters were 11000-, 300-, and 40-fold lower at 2-, 4-, and 7 days post-contact, respectively (Supplementary Fig. 5b). The results suggest that ∆3678 virus could be transmitted in hamsters, although at a much lower efficiency than the WT virus.

### Genetic stability of ∆3678 SARS-CoV-2 on Vero-E6 and Vero-E6-TMPRSS2 cells

We initially examined the genetic stability of ∆3678 virus by continuously culturing the virus for five rounds on Vero-E6 cells. Three independent, parallel passaging experiments were performed to assess the consistency of adaptive mutations (Supplementary Fig. 6a). The passage 5 (P5) virus developed bigger plaques than the original P0 virus (Supplementary Fig. 6b). Full-genome sequencing of the P5 viruses identified an H655Y substitution in the spike protein and a 675-679 QTQTN spike deletion from all three passage series (Supplementary Fig. 6c). The substitution and deletion are located immediately upstream of the furin cleavage site between the spike 1 and 2 subunits. Since previous studies reported that culturing of SARS-CoV-2 on Vero-E6 cells expressing serine protease TMPRSS2 (Vero-E6-TMPRSS2) could eliminate such mutations/deletions^30–32^, we passaged the ∆3678 virus on the Vero-E6-TMPRSS2 cells for 10 rounds. Indeed, no spike mutations were observed in the P10 virus. In addition, all the engineered TRS mutations were also retained in the P10 virus. These results suggest that Vero-E6-TMPRSS2 cells could be used for the production of ∆3678 vaccine candidates.

### Contribution of individual ORFs to the attenuation of ∆3678 virus

**Fig. 5:**
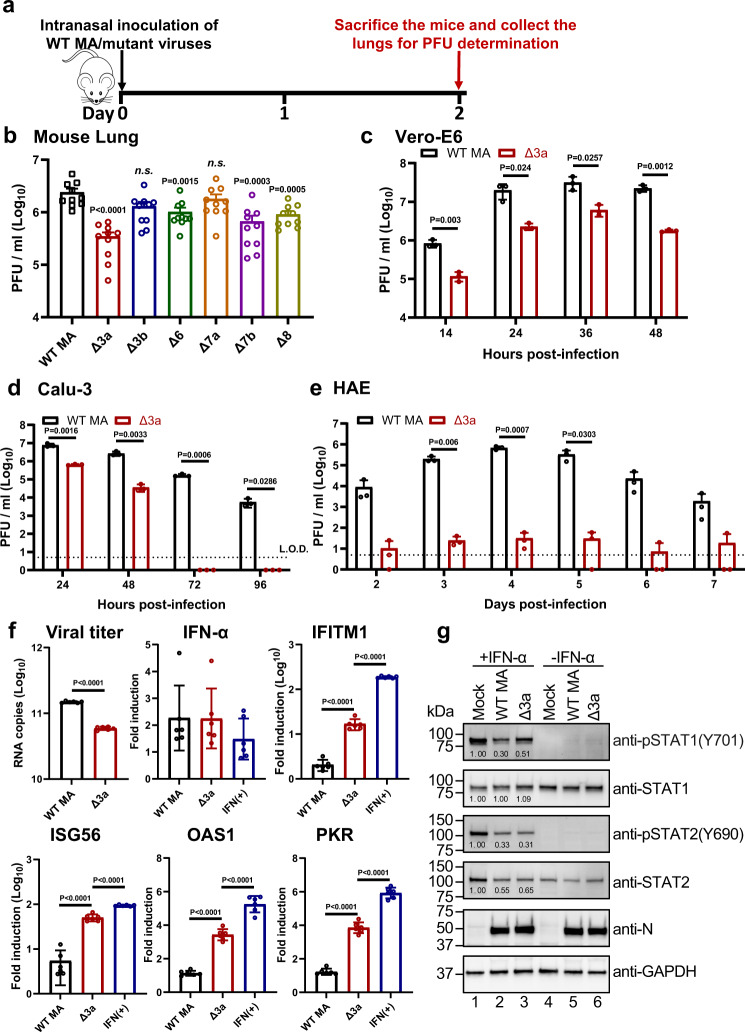
ORF3a deletion is mainly responsible for the attenuation of Δ3678 virus through interfering with STAT1 phosphorylation during type-I interferon signaling. **a**, **b** Analysis of individual ORFs in BALB/c mice. **a** Experimental design. **b** Lung viral loads from mice infected with different ORF-deletion viruses. Dots represent individual animals (*n* = 10). The mean ± standard error of the mean is presented. A non-parametric two-tailed Mann–Whitney test was used to determine the statistical difference between the WT and ORF-deletion groups. *P* values were adjusted using the Bonferroni correction to account for multiple comparisons. Differences were considered significant if *p* < 0.0083. **c**–**e** Replication kinetics of Δ3a virus in Vero-E6 (**c**), Calu-3 (**d**), and HAE (**e**) cells. Dots represent individual biological replicates (*n* = 3). The values represent the mean ± standard deviation. An unpaired two-tailed *t* test was used to determine significant differences. **f** ORF3a deletion increases ISG expression in Δ3a-infected A549-hACE2 cells. Viral RNA copies and mRNA levels of IFN-α, IFITM1, ISG56, OAS1, PKR, and GAPDH were measured by RT-qPCR. GAPDH was used to normalize the ISG mRNA levels. The mRNA levels are presented as fold induction over mock samples. As a positive control, uninfected cells were treated with 1000 units/ml IFN-α for 24 h. Dots represent individual biological replicates (*n* = 6). The data represent the mean ± standard deviation. An unpaired two-tailed *t* test was used to determine significant differences between Δ3a and WT-MA or IFN(+) groups. *P* values were adjusted using the Bonferroni correction to account for multiple comparisons. Differences were considered significant if *p* < 0.025. **g** ORF3a suppresses type-I interferon by reducing STAT1 phosphorylation. A549-hACE2 cells were pre-treated with or without 1000 units/ml IFN-α for 6 h. The cells were then infected with WT-MA or Δ3a virus at an MOI of 1 for 1 h. The infected cells were washed twice with PBS, continuously cultured in fresh media with or without 1000 units/ml IFN-α, and analyzed by Western blot at 24 h post-infection. The values under the bands indicate the ratios of densitometries of WT-MA and Δ3a over the MOCK. **b**–**g** Source Data are provided as a Source Data file.

To define the role of each ORF in attenuating ∆3678 virus, we prepared a panel of mutant viruses in the backbone of a mouse-adapted SARS-CoV-2 (MA-SARS-CoV-2) that can robustly infect BALB/c mice^33^. Each mutant virus contained a single accessory gene deletion, including ∆3a, ∆3b, ∆6, ∆7a, ∆7b, or ∆8 (Supplementary Fig. 7a). Among all the individual deletion mutants, ∆3a virus developed the smallest plaques on Vero-E6 cells (Supplementary Fig. 7b). The biological importance of each deleted gene was analyzed by viral replication in the lungs after intranasal infection of BALB/c mice (Fig. 5a). On day 2 post-infection, deletion of ∆3a, ∆3b, ∆6, ∆7b, or ∆8 reduced viral loads in lungs, among which ∆3a exhibited the largest reduction (Fig. 5b). To further confirm the critical role of ∆3a in viral attenuation, we compared the replication kinetics between ∆3a and WT-MA-SARS-CoV-2 on Vero-E6 (Fig. 5c), Calu-3 (Fig. 5d), and HAE cultures (Fig. 5e). The replication of ∆3a was significantly more attenuated in immune-competent Calu-3 and HAE cells than in interferon-deficient Vero-E6 cells (Fig. 5c–e). Taken together, the results indicate that ∆3a played a major role in attenuating the ∆3678 virus, possibly through the type-I interferon pathway.

### ORF3a antagonizes type-I interferon signaling by inhibiting STAT1 phosphorylation

To define the mechanism of ∆3a-mediated viral attenuation, we infected human lung A549 cells expressing the human ACE2 receptor (A549-hACE2) with ∆3a or WT-MA-SARS-CoV-2. Although the replication of ∆3a was lower than that of the WT virus, comparable levels of IFN-α RNA were produced (Fig. 5f). Significantly higher levels of interferon-stimulating genes (ISGs), such as IFITM1, ISG56, OAS1, and PKR, were detected in the ∆3a virus-infected cells than in the WT virus-infected cells (Fig. 5f), suggesting a role of ORF3a in suppressing type-I interferon signaling. To further bolster this observation, we treated the A549-hACE cells with IFN-α followed by ∆3a or WT virus infection. Western blot analysis showed that the phosphorylation of STAT1 was more efficient in the ∆3a-infected cells than the WT-infected cells, whereas no difference in STAT2 phosphorylation was observed (Fig. 5g). Thus, the results indicate that ORF3a protein suppresses STAT1 phosphorylation during type-I interferon signaling.

### An mNG reporter ∆3678 virus for neutralization and antiviral testing

**Fig. 6:**
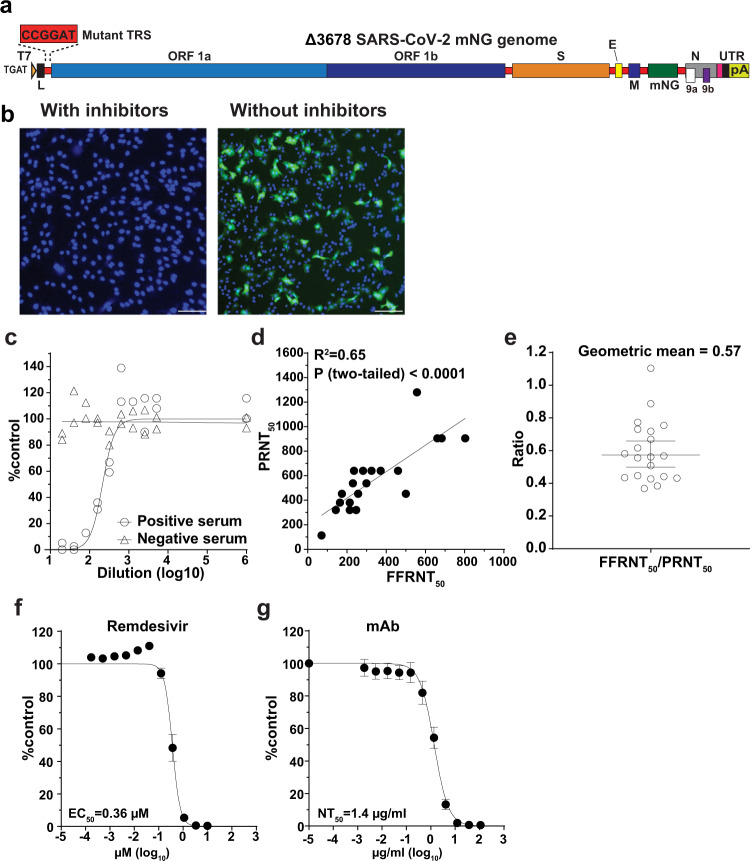
Development of mNG Δ3678 virus for high-throughput neutralization and antiviral testing. **a** Genome structure of mNG Δ3678 SARS-CoV-2. The mNG gene was inserted between M and N genes. **b** mNG foci of A549-ACE2 cells that were infected with mNG Δ3678 SARS-CoV-2 in the presence and absence of 10 µM inhibitor (remdesivir) for 24 h. Scale bar, 100 µm. **c** Representative FFRNT neutralization curves for a COVID-19 antibody-positive and -negative serum. **d** Correlation between FFRNT_50_ with PRNT_50_ values of 20 COVID-19 convalescent sera. The Pearson correlation (two-sided) efficiency and *P* value are shown. **e** Scatter-plot of FFRNT_50_/PRNT_50_ ratios. The geometric mean is shown. The error bar indicates the 95% confidence interval of the geometric mean. **f** Antiviral response curve of remdesivir against mNG Δ3678 SARS-CoV-2. The calculated 50% effective concentration (EC_50_) is indicated. **g** The Dose response curve of a monoclonal antibody IgG14. A549-hACE2 cells were infected with mNG Δ3678 virus in the presence of different concentrations of IgG14. The mNG signals at 24 h post-infection were used to calculate the NT_50_. **f**, **g** Error bars indicate the mean ± standard deviations from four technical replicates. **c**–**g** Source Data are provided as a Source Data file.

The in vitro and in vivo attenuation results suggest that ∆3678 virus may serve as a research tool for BSL-2 use. To further develop this tool, we engineered an mNG gene (driven by its own TRS sequence) between the M and N genes of the ∆3678 genome, resulting in mNG ∆3678 virus (Fig. 6a). The mNG gene was stably retained after continuously culturing the reporter virus on Vero-E6-TMPRSS2 cells for 10 rounds, as indicated by (i) next-generation sequencing (NGS) result and (ii) mNG-positive cells. For high-throughput neutralization testing, we developed the mNG ∆3678 virus into a fluorescent focus reduction neutralization test (FFRNT) in a 96-well format. When infecting the cells, the mNG ∆3678 developed fluorescent foci that could be quantified by high content imaging (Fig. 6b). Figure 6c shows the FFRNT curves for one COVID-19 convalescent positive serum and one negative serum. To validate the FFRNT assay, we tested 20 convalescent sera against the mNG ∆3678 virus. For comparison, the same serum panel was tested against the WT SARS-CoV-2 (without mNG) using the gold-standard plaque-reduction neutralization test (PRNT)^27^. The 50% reduction neutralization titers (NT_50_) correlated well between the FFRNT and PRNT assays (Fig. 6d). The geometric mean of FFRNT_50_/PRNT_50_ ratio was 0.57 for the tested serum panel (Fig. 6e). Next, we developed the reporter mNG ∆3678 virus into a high-throughput antiviral assay. Treatment of the mNG ∆3678 virus-infected A549-hACE2 cells with remdesivir diminished the appearance of mNG-positive cells (Fig. 6b, f). Dose-responsive antiviral curves were reliably obtained for a monoclonal antibody (Fig. 6g). Overall, the results demonstrate that mNG ∆3678 virus could be used for high-throughput neutralization and antiviral testing.

## Discussion

We have developed ∆3678 SARS-CoV-2 as a potential live-attenuated vaccine candidate. The ∆3678 virus could replicate to titers >5.6 × 10^6^ PFU/ml on interferon-incompetent Vero-E6 cells (Fig. 1c), making large-scale production feasible in this vaccine manufacture-approved cell line or its derivative Vero-E6-TMPRSS2 cells. In contrast, the ∆3678 virus was highly attenuated when infecting immune-competent cells, as evidenced by the 7500-fold reduction in viral replication of WT virus on human primary HAE cells (Fig. 1e). In both hamster and K18-hACE2 mouse models, the ∆3678 infections did not cause significant weight loss or death at the highest tested infection dose [10^6^ PFU for hamsters (Fig. 2b) and 4 × 10^4^ PFU for K18-hACE2 mice (Fig. 4c, g)], whereas the WT virus caused weight loss and death at a much lower infection dose (>4 × 10^2^ PFU for K18-hACE2 mice; Fig. 4b, f). Analysis of individual ORF deletion viruses identified ORF3a as a major accessory protein responsible for the attenuation of the ∆3678 virus (Fig. 5b); this conclusion was further supported by the observation that the addition of ∆3a to the ∆678 virus significantly increased the attenuation of ∆3678 replication (Fig. 1c–f). Our results are supported by a recent study reporting that ∆3a SARS-CoV-2 and, to a less extend, ∆6 SARS-CoV-2 were attenuated in the K18-hACE2 mice^34^. Mechanistically, we found that ORF3a protein antagonized the innate immune response by blocking STAT1 phosphorylation during type-I interferon signaling. Thus, the deletion of ORF3a conferred SARS-CoV-2 more susceptible to type-I interferon suppression. Our results are supported by previous reports that expression of SARS-CoV-2 ORF3a alone antagonized type-I interferon signaling^19,35^. The ORF3a protein was recently shown to form a dimer in the cell membrane with an ion channel activity^36^, which may account for its role in cell membrane rearrangement, inflammasome activation, and apoptosis^37–39^. Whether the ion channel activity of ORF3a is required for the inhibition of STAT1 phosphorylation remains to be determined.

The attenuated ∆3678 viruses may be pivoted for a veterinarian vaccine. SARS-CoV-2 can infect a variety of animal species, among which cats, ferrets, fruit bats, hamsters, minks, raccoon dogs, and white-tailed deers were reported to spread the infection to other animals of the same species^40–44^, and potentially spillback to humans. A live-attenuated ∆3678 vaccine may be useful for the prevention and control of SARS-CoV-2 on mink farms^45^. Since zoonotic coronaviruses may recombine with the live-attenuated vaccine in immunized animals, we engineered the ∆3678 viruses with mutated TRS to eliminate the possibility of recombination-mediated emergence of WT or replicative chimeric coronaviruses (Fig. 1a). This mutated TRS approach was previously shown to attenuate SARS-CoV and to prevent reversion of the WT virus^12,13^. Given the continuous emergence of SARS-CoV-2 variants, we could update the vaccine antigen by swapping the variant spike glycoproteins into the current ∆3678 virus backbone. This approach will enable a rapid modification of the vaccine spike to match the newly emerged variants.

The attenuated ∆3678 virus could serve as a research tool that might be used at BSL-2. Using mNG as an example, we developed an mNG ∆3678 virus for high-throughput testing of antibody neutralization and samll molecule inhibitors (Fig. 6). Depending on research needs, other reporter genes, such as luciferase or other fluorescent genes, could be engineered into the system. This high-throughput assay can be modified for testing neutralization against different variants by swapping the variant spike genes into the ∆3678 backbone. The approach has been successfully used to study vaccine-elicited neutralization against variants in the context of complete SARS-CoV-2^46–48^. Finally, our in vitro and in vivo attenuation results support the possible use of the ∆3678 virus at BSL-2. If further attenuation is needed, more mutations, such as inactivating the NSP16 2’-O methyltransferase activity^49^ or 7-mucleotide TRS mutation (TRS7)^12,13^, can be rationally engineered into the ∆3678 virus.

One limitation of the current study is that we have not defined the attenuation mechanisms of the ORF 3b, 6, 7b, or 8 deletion, even though they reduced the lung viral loads in the K18-hACE2 mice (Fig. 5b). SARS-CoV-2 ORF8 protein was recently reported to contain a histone mimic that could disrupt chromatin regulation and enhance viral replication^50^. Truncations or deletions of ORF7b and ORF8 were reported in SARS-CoV-2 clinical isolates^51,52^. Future studies are needed to understand the molecular functions of OFR 3b, 6, and 7b proteins. Another limitation of the current study is the lack of T cell and mucosal immunity analyses; studies are ongoing to address these key protective immunities after ∆3678 infection. Nevertheless, our available results indicate that ∆3678 virus could serve as a potential live-attenuated vaccine candidate and as an experimental system that can likely be performed at BSL-2 for COVID-19 research and countermeasure development.

## Methods

### Ethics statement

Hamster and mouse studies were performed in accordance with the guidance for the Care and Use of Laboratory Animals of the University of Texas Medical Branch (UTMB). The protocol was approved by the Institutional Animal Care and Use Committee (IACUC) at UTMB. The animals were maintained at macroenvironmental temperature and humidity ranges of 65 to 75 °F and 40% to 60%, respectively with a 12 light/12 dark light cycle. All the animal operations were performed under anesthesia by isoflurane to minimize animal suffering. The convalescent sera from COVID-19 patients (confirmed by the molecular tests with FDA’s Emergency Use Authorization) were leftover specimens and completely de-identified from patient information. The use of human COVID-19 sera was reviewed and approved by the UTMB Institutional Review Board (IRB#: 20-0070). The serum specimens were heat-inactivated at 56 °C for 30 min before testing.

### Animals and Cells

The Syrian golden hamsters (HsdHan:AURA strain) were purchased from Envigo (Indianapolis, IN). K18-hACE2 mice were purchased from the Jackson Laboratory (Bar Harbor, ME). BALB/c mice were purchased from Charles River Laboratories (Wilmington, MA). African green monkey kidney epithelial Vero-E6 cells (laboratory-passaged derivatives from ATCC CRL-1586) were grown in Dulbecco’s modified Eagle’s medium (DMEM; Gibco/Thermo Fisher, Waltham, MA, USA) with 10% fetal bovine serum (FBS; HyClone Laboratories, South Logan, UT) and 1% antibiotic/streptomycin (P/S, Gibco). Vero-E6-TMPRSS2 cells were purchased from SEKISUI XenoTech, LLC (Kansas City, KS) and maintained in 10% fetal bovine serum (FBS; HyClone Laboratories, South Logan, UT) and 1% P/S and 1 mg/ml G418 (Gibco). The A549-hACE2 cells that stably express hACE2 were generously provided by Shinji Makino^53^ and grown in the DMEM supplemented with 10% fetal bovine serum, 1% P/S and 1% 4-(2-hydroxyethyl)-1-piperazineethanesulfonic acid (HEPES; Thermo Fisher Scientific) and 10 μg/mL Blasticidin S. Human lung adenocarcinoma epithelial Calu-3 cells (ATCC, Manassas, VA, USA) were maintained in a high-glucose DMEM containing sodium pyruvate and GlutaMAX (Gibco) with 10% FBS and 1% penicillin/streptomycin at 37 °C with 5% CO_2_. The EpiAirway system is a primary human airway 3D tissue model purchased from MatTek Life Science (Ashland, MA, USA). All cells were maintained at 37 °C with 5% CO_2_. All cell lines were verified and tested negative for mycoplasma.

### Generation of SARS-CoV-2 mutant viruses

(1) Generate mutant viruses with accessory ORF deletions. The ORF 6, 7, and 8 deletions (∆678) and ORF 3, 6, 7, and 8 deletions (∆3678) were constructed by overlap PCR using an infection clone of USA-WA1/2020 SARS-CoV-2^14^. The ∆3a, ∆3b, ∆6, ∆7a, ∆7b, ∆8 mutants were engineered into an infection clone of a mouse-adapted SARS-CoV-2 (MA-SARS-CoV-2)^33^ using a standard molecular cloning protocol. For generating ∆ORF6 and ∆ORF8 mutants, an overlapping PCR strategy was used to delete the ORF and the upstream transcriptional regulatory sequence (TRS). For generating ∆ORF3a and ∆ORF7a mutants, the complete ORF3 and ORF7 were replaced with the ORF3b- and ORF7b-coding sequence, respectively. For generating ∆ORF3b mutant, several nonsense mutations were introduced into MA-SARS-CoV-2 to disrupt the initiation codon of ORF3b without affecting the translation of ORF3a (i.e., the change of sequence from wild-type TATGATG to mutant TA**C**GA**C**G; ORF3b-coding sequence is underlined and the two mutated nucleotides are in bold). For producing ∆ORF7b mutant, the initiation codon of ORF7b was disrupted, and the ORF7b gene was deleted from the fifth nucleotide position onward. (2) Generate reporter viruses with accessory ORF deletions. The mNG WT and mNG ∆3678 SARS-CoV-2s were generated by engineering the mNeonGreen (mNG) gene into the ORF7 position of the WT and ∆3678 viruses. The mutant infectious clones were assembled by in vitro ligation of contiguous DNA fragments following the protocol previously described^54^. In vitro transcription was then performed to synthesize genomic RNA. For recovering the viruses, the RNA transcripts were electroporated into Vero-E6 cells. The viruses from electroporated cells were harvested at 40 h post-electroporation and served as P0 stocks. All viruses were passaged once on Vero-E6 cells, resulting in P1 stocks for subsequent experiments. The P1 virus stocks were sequenced to confirm no undesired mutations. Only the passage 1 virus stocks with confirmed sequences were used in the study. Viral titers were determined by plaque assay on Vero-E6 cells. All virus preparation and experiments were performed in a BSL-3 facility. Viruses and plasmids are available from the World Reference Center for Emerging Viruses and Arboviruses (WRCEVA) at the University of Texas Medical Branch.

### RNA extraction, RT-PCR, and cDNA sequencing

Cell culture supernatants or clarified tissue homogenates were mixed with a five-fold excess of TRIzol™ LS Reagent (Thermo Fisher Scientific, Waltham, MA). Viral RNAs were extracted according to the manufacturer’s instructions. The extracted RNAs were dissolved in 20 μl nuclease-free water. For sequence validation of mutant viruses, 2 µl of RNA samples were used for reverse transcription by using the SuperScript™ IV First-Strand Synthesis System (Thermo Fisher Scientific) with random hexamer primers. Nine DNA fragments flanking the entire viral genome were amplified by PCR. The resulting DNAs were cleaned up by the QIAquick PCR Purification Kit, and the genome sequences were determined by Sanger sequencing at GENEWIZ (South Plainfield, NJ).

### Viral infection of cell lines

Approximately 3 × 10^5^ Vero-E6 or Calu-3 cells were seeded onto each well of 12-well plates and cultured at 37 °C, 5% CO_2_ for 16 h. SARS-CoV-2 WT or mutant viruses were inoculated onto Vero-E6 and Calu-3 cells at an MOI of 0.01 and 0.1, respectively. After 2 h infection at 37 °C with 5% CO_2_, the cells were washed with DPBS 3 times to remove any detached virus. One milliliter of culture medium was added to each well for the maintenance of the cells. At each time point, 100 µl of culture supernatants were collected for detection of virus titer, and 100 µl of fresh medium was added to each well to replenish the culture volume. The cells were infected in triplicate for each group of viruses. All samples were stored at −80 °C until analysis.

### Viral infection in a primary human airway cell culture model

The EpiAirway system is a primary human airway 3D mucociliary tissue model consisting of normal, human-derived tracheal/bronchial epithelial cells. For viral replication kinetics, WT or mutant viruses were inoculated onto the culture at an indicated MOI in DPBS. After 2 h infection at 37 °C with 5% CO_2_, the inoculum was removed, and the culture was washed three times with DPBS. The infected epithelial cells were maintained without any medium in the apical well, and the medium was provided to the culture through the basal well. The infected cells were incubated at 37 °C, 5% CO_2_. From 1–7 days, 300 μl of DPBS were added onto the apical side of the airway culture and incubated at 37 °C for 30 min to elute the released viruses. All virus samples in DPBS were stored at −80 °C.

### Quantitative real-time RT-PCR assay

RNA copies of SARS-CoV-2 samples and mRNA levels of IFN-α, IFITM1, ISG56, OAS1, PKR, and GAPDH were determined by quantitative real-time RT-PCR (RT-qPCR) assays. The RT-qPCR assays used the iTaq SYBR Green One-Step Kit (BioRad) on the LightCycler 480 system (Roche, Indianapolis, IN) following the protocol: (1) 50 °C, 10 min; 95 °C, 5 min; (2) 95 °C, 15 s; 60 °C, 30 s; 40 cycles; (3) 95 °C, 15 s; 60 °C to 95 °C, increment 0.5 °C, 0.05 s. The primers are listed in Supplementary Table 1. The absolute quantification of viral RNA was determined by a standard curve method using an RNA standard (in vitro transcribed 3,840-nucleotide viral RNA representing nucleotide positions 26,044 to 29,883 of the SARS-CoV-2 genome). The housekeeping gene GAPDH was used to normalize all mRNA levels.

### Hamster immunization and challenge assay

Four- to six-week-old male golden Syrian hamsters, strain HsdHan:AURA (Envigo, Indianapolis, IN), were immunized intranasally with 100 µl WT virus (10^6^ PFU, *n* = 20) or ∆3678 mutant virus (10^6^ PFU, *n* = 20). Animals received DMEM media (supplemented with 2% FBS and 1% penicillin/streptomycin) served as Mock group (*n* = 20). On day 28, the animals were challenged with 10^5^ PFU of WT SARS-CoV-2. The animals were weighed and monitored for signs of illness daily. Nasal washes and oral swabs were collected in 400 µl sterile DPBS and 1 ml DMEM media at indicated time points. For organ collection, animals were humanely euthanized on days 2, 30, and 32, tracheae and lungs were harvested and placed in a 2-ml homogenizer tube containing 1 ml of DMEM media. On day 49, animals were humanely euthanized for blood collection, serum were then isolated for neutralization titer (NT_50_) detection. The NT_50_ values were determined using an mNG USA-WA1/2020 SARS-CoV-2 as previously reported^27^.

To test if lower dose immunization could achieve protection, hamsters were immunized with 10^2^, 10^3^, 10^4^, or 10^5^ PFU of ∆3678 virus. Nasal washes, oral swabs, and organs were collected at indicated time points. The animals were weighed and monitored for signs of illness daily.

### Hamster transmission assay

Hamster transmission assay was performed per our previous protocol^28^. Briefly, hamsters were immunized intranasally with 10^6^ PFU ∆3678 mutant virus (*n* = 5). Animals who received DMEM media served as a mock group (*n* = 5). On day 28 post-immunization, the animals were challenged with 10^5^ PFU of WT SARS-CoV-2. One day later, one infected donor animal was co-housed with one naive animal for 8 h (5 pairs for mock group, 5 pairs for ∆3678 group). After the 8-h contact, the donor and recipient animals were separated and maintained in individual cages. Nasal washes were collected at indicated time points. On day 42, animals were humanely euthanized for blood collection.

### Mouse infection of ∆3678 virus

Eight- to ten-week-old K18-hACE2 female mice were intranasally infected with 50 µl different doses of WT or ∆3678 virus (4, 40, 400, 4000, 40,000 PFU, *n* = 10 per dose). Animals received DMEM media and served as a mock group. Lungs were collected on days 2, 4, and 7 post-infection and placed in a 2-ml homogenizer tube containing 1 ml of DMEM media for tissue homogenate. Animals were weighed and monitored for signs of illness daily and were sacrificed on day 14.

To define the role of each ORF in attenuating ∆3678 virus, 8- to 10-week-old BALB/c female mice were intranasally infected with 50 µl WT mouse-adapted-SARS-CoV-2 (10^4^ PFU, *n* = 10) or ∆3a, ∆3b, ∆6, ∆7a, ∆7b, ∆8 virus (10^4^ PFU, *n* = 10 per virus). On day 2 post-infection, animals were humanely euthanized for lung collection.

### Histopathology

Hamsters were euthanized with carbon dioxide (CO_2_) inhalation and necropsy was performed. The lungs were inspected for gross lesions and representative portions of the lungs were collected in 10% buffered formalin for histology. Formalin-fixed tissues were processed per a standard protocol, 4 μm-thick sections were cut and stained with hematoxylin and eosin (HE). The slides were imaged in a digital scanner (Leica Aperio LV1). Lung sections were examined under light microscopy using an Olympus CX43 microscope for the extent of inflammation, size of inflammatory foci, and changes in alveoli, alveolar septa, airways, and blood vessels. The blinded tissue sections were semi-quantitatively scored for pathological lesions. The criteria for histopathology scoring are presented in Supplementary Table 2.

### Plaque assay

Approximately 1.2 × 10^6^ Vero-E6 cells were seeded to each well of 6-well plates and cultured at 37 °C, 5% CO_2_ for 16 h. Virus was serially diluted in DMEM with 2% FBS and 200 µl diluted viruses were transferred onto the monolayers. The viruses were incubated with the cells at 37 °C with 5% CO_2_ for 1 h. After the incubation, overlay medium was added to the infected cells per well. The overlay medium contained DMEM with 2% FBS, 1% penicillin/streptomycin, and 1% sea-plaque agarose (Lonza, Walkersville, MD). After a 2.5-day incubation, the plates were stained with neutral red (Sigma-Aldrich, St. Louis, MO) and plaques were counted on a lightbox. The organ titers of hamsters were calculated to PFU per gram. The detection limits of the plaque assay were indicated in the figures.

### Genetic stability of ∆3678 SARS-CoV-2

The P0 ∆3678 SARS-CoV-2 was continuously cultured for five rounds on Vero-E6 cells. Three independent passaging experiments were performed to assess the consistency of adaptive mutations. The P5 viral RNAs from three independent replicates were extracted and subjected to RT-PCR. Whole-genome sequencing was performed on RT-PCR products. The mutations that occurred in the P5 Δ3678 viruses were analyzed. To increase the genetic stability of spike gene, we continuously passaged the Δ3678 virus on Vero-E6-TMPRSS2 cells for ten rounds. The P10 viruses were subjected to complete genome sequencing.

### ORF3a-mediated suppression of type-I interferon signaling

A549-hACE2 cells were infected with WT or ΔORF3a SARS-CoV-2 at an MOI of 1 for 1 h, after which the cells were washed twice with PBS and cultured in a fresh medium. Intracellular RNAs were harvested at 24 h post-infection. Viral RNA copies and mRNA levels of IFN-α, IFITM1, ISG56, OAS1, PKR, and GAPDH were determined by quantitative RT-PCR. The housekeeping gene GAPDH was used to normalize mRNA levels and the mRNA levels are presented as fold induction over mock samples. As a positive control, uninfected cells were treated with 1000 units/ml IFN-α for 24 h.

### Suppression of STAT1 phosphorylation by ORF3a protein

A549-hACE2 cells were pre-treated with 1000 units/ml IFN-α for 6 h. Mock-treated cells were used as a control. Cells were infected with WT or ΔORF3 SARS-CoV-2 at an MOI 1 for 1 h. Inoculums were removed; cells were washed twice with PBS; fresh media with or without 1000 units/ml IFN-α were added. Samples were collected at 24 h post-infection by using 2× Laemmli buffer (BioRad, #1610737) and analyzed by Western blot. Recombinant human α-interferon (IF007) was purchased from Millipore (Darmstadt, Germany). Anti-STAT1 (14994 S, 1:1000), anti-pSTAT1 (Y701) (7649 S, 1:1000), anti-STAT2 (72604 S, 1:1000), anti-pSTAT2 (Y690) (88410 S, 1:1000) antibodies were from Cell Signaling Technology (Danvers, MA); anti-GAPDH (G9545, 1:1000) antibodies were from Sigma-Aldrich; SARS-CoV-2 (COVID-19) nucleocapsid antibody (NB100-56576, 1:1000) were from Novus Biologicals (CO, USA).

### Fluorescent focus reduction neutralization test (FFRNT)

Neutralization titers of COVID-19 convalescent sera were measured by a fluorescent focus reduction neutralization test (FFRNT) using mNG Δ3678 SARS-CoV-2. Briefly, Vero-E6 cells (2.5 × 10^4^) were seeded in each well of black μCLEAR flat-bottom 96-well plate (Greiner Bio-one™). The cells were incubated overnight at 37 °C with 5% CO2. On the following day, each serum was 2-fold serially diluted in the culture medium with the first dilution of 1:20. Each serum was tested in duplicates. The diluted serum was incubated with 100-150 fluorescent focus units (FFU) of mNG SARS-CoV-2 at 37 °C for 1 h (final dilution range of 1:20 to 1:20,480), after which the serum-virus mixtures were inoculated onto the pre-seeded Vero-E6 cell monolayer in 96-well plates. After 1 h infection, the inoculum was removed and 100 μl of overlay medium (DMEM supplemented with 0.8% methylcellulose, 2% FBS, and 1% P/S) was added to each well. After incubating the plates at 37 °C for 16 h, raw images of mNG fluorescent foci were acquired using CytationTM 7 (BioTek) armed with 2.5× FL Zeiss objective with widefield of view and processed using the software settings (GFP [469,525] threshold 4000, object selection size 50–1000 µm). The foci in each well were counted and normalized to the non-serum-treated controls to calculate the relative infectivities. The curves of the relative infectivity versus the serum dilutions (log10 values) were plotted using Prism 9 (GraphPad). A nonlinear regression method with log (inhibitor) vs. response-variable slope (four parameters) model (bottom and top parameters were constrained to 0 and 100, respectively) was used to determine the dilution fold that neutralized 50% of mNG SARS-CoV-2 (defined as FFRNT_50_) in GraphPad Prism 9. Each serum was tested in duplicates.

### Antiviral testing

A549-hACE2 cells were used to evaluate the efficacy of a monoclonal antibody IgG14 and antiviral drug remdesivir. The sources of IgG14 and remdesivir were previously reported^15,55,56^. Briefly, A549-hACE2 cells (1.2 × 10^4^) were seeded in each well of black μCLEAR flat-bottom 96-well plate (Greiner Bio-one™). The cells were incubated overnight at 37 °C with 5% CO_2_. For antibody testing, on the following day, IgG14 was 3-fold serially diluted and incubated with mNG Δ3678 at 37 °C for 1 h, after which the antibody-virus mixtures were inoculated into the 96-well plates that were pre-seeded A549-hACE2 cells. For antiviral testing, remdesivir was 3-fold serially diluted in DMSO and further diluted as 100 folds in the culture medium containing mNG Δ3678 virus. Fifty µl of the compound-virus mixture were immediately added to the cells at a final MOI of 1.0. At 24 h post-infection, 25 μl of Hoechst 33342 Solution (400-fold diluted in Hank’s Balanced Salt Solution; Gibco) were added to each well to stain the cell nucleus. The plate was sealed with Breath-Easy sealing membrane (Diversified Biotech), incubated at 37 °C for 20 min, and quantified for mNG fluorescence on CX5 imager (Thermo Fisher Scientific). The raw images (2 × 2 montage) were acquired using ×4 objective. The total cells (indicated by nucleus staining) and mNG-positive cells were quantified for each well. Infection rates were determined by dividing the mNG-positive cell number by the total cell number. Relative infection rates were obtained by normalizing the infection rates of treated groups to those of no-treated controls. The curves of the relative infection rates versus the concentration were plotted using Prism 9 (GraphPad). A nonlinear regression method was used to determine the concentration of antiviral that suppress 50% of mNG fluorescence (EC_50_). The experiment was tested in quadruplicates.

### Statistics

Hamsters and mice were randomly allocated into different groups. The investigators were not blinded to allocation during the experiments or to the outcome assessment. No statistical methods were used to predetermine sample size. Descriptive statistics have been provided in the figure legends. For in vitro replication kinetics, Kruskal–Wallis analysis of variance was conducted to detect any significant variation among replicates. If no significant variation was detected, the results were pooled for further comparison. Differences between continuous variables were assessed with a non-parametric Mann–Whitney test. The PFU and genomic copies were analyzed using an unpaired two-tailed *t* test. The weight loss data were shown as mean ± standard deviation and statistically analyzed using two-factor analysis of variance (ANOVA) with Tukey’s post hoc test. The animal survival rates were analyzed using a mixed-model ANOVA using Dunnett’s test for multiple comparisons. Analyses were performed in Prism version 9.0 (GraphPad, San Diego, CA).

### Reporting summary

Further information on research design is available in the Nature Research Reporting Summary linked to this article.

## Supplementary information

Supplementary Information

Reporting Summary

## Data Availability

The mNG reporter ∆3678 SARS-CoV-2 will be deposited to the World Reference Center for Emerging Viruses and Arboviruses (https://www.utmb.edu/wrceva) at UTMB for distribution. Any other information is available upon request. Source data are provided with this paper.

## Source data

Source Data
