# Publisher Correction: A live-attenuated SARS-CoV-2 vaccine candidate with accessory protein deletions

**DOI:** 10.1038/s41467-022-33878-6

**Published:** 2022-10-13

**Authors:** Yang Liu, Xianwen Zhang, Jianying Liu, Hongjie Xia, Jing Zou, Antonio E. Muruato, Sivakumar Periasamy, Chaitanya Kurhade, Jessica A. Plante, Nathen E. Bopp, Birte Kalveram, Alexander Bukreyev, Ping Ren, Tian Wang, Vineet D. Menachery, Kenneth S. Plante, Xuping Xie, Scott C. Weaver, Pei-Yong Shi

**Affiliations:** 1 Department of Biochemistry and Molecular Biology, University of Texas Medical Branch, Galveston, TX USA; 2 Department of Microbiology and Immunology, University of Texas Medical Branch, Galveston, TX USA; 3 Institute for Human Infections and Immunity, University of Texas Medical Branch, Galveston, TX USA; 4 Department of Pathology, University of Texas Medical Branch, Galveston, TX USA; 5 Galveston National Laboratory, Galveston, TX USA; 6 World Reference Center for Emerging Viruses and Arboviruses, University of Texas Medical Branch, Galveston, TX USA; 7 Sealy Institute for Vaccine Sciences, University of Texas Medical Branch, Galveston, TX USA; 8 Sealy Institute for Drug Discovery, University of Texas Medical Branch, Galveston, TX USA; 9 Sealy Center for Structural Biology & Molecular Biophysics, University of Texas Medical Branch, Galveston, TX USA

**Keywords:** Live attenuated vaccines, SARS-CoV-2

**Correction to**: *Nature Communications* 10.1038/s41467-022-31930-z, published online 27 July 2022

The original version of this Article contained an error in Fig. 3h, in which the numbers indicating days of infection and sample collection were incorrectly placed along the timeline. This has been corrected in both the PDF and HTML versions of the Article.

