# Supplementary material for: A live-attenuated SARS-CoV-2 vaccine candidate with accessory protein deletions
Source: Nat Commun. 2022 Jul 27;13:4337. doi: 10.1038/s41467-022-31930-z (PMC9326133; doi:10.1038/s41467-022-31930-z)
Supplement: Supplementary file 1 — Supplementary Information [file 41467_2022_31930_MOESM1_ESM.pdf]

# A live-attenuated SARS-CoV-2 vaccine candidate with accessory protein deletions

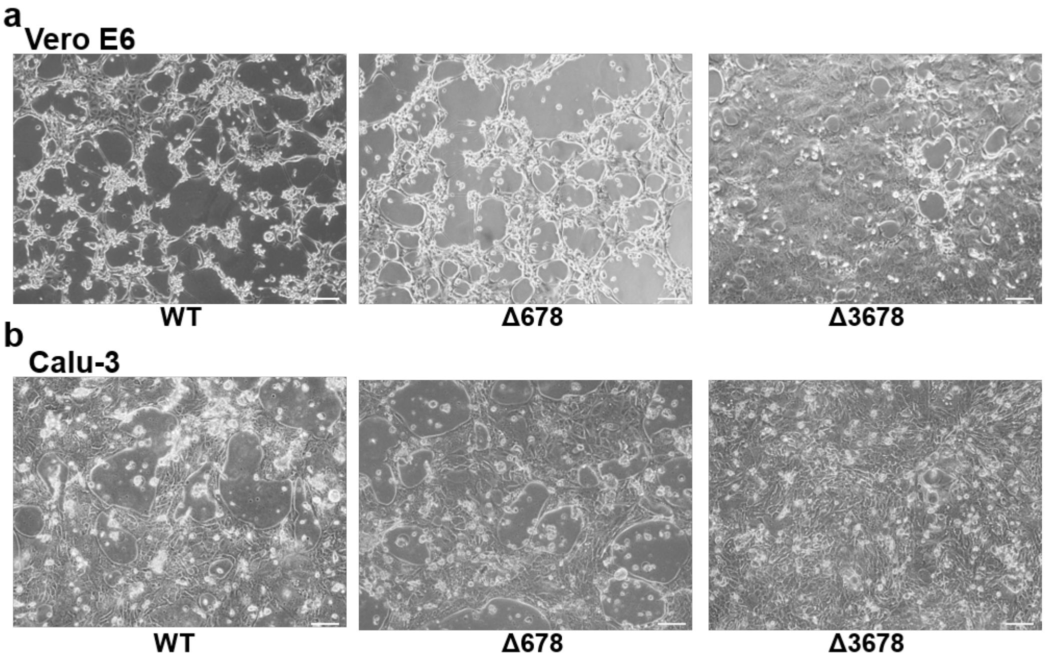


**Supplementary Fig. 1. Brightfield images of the cytopathic effects of WT, Δ678, and Δ3678 SARS-CoV-2-infected Vero-E6 and Calu-3 cells.** The Vero-E6 and Calu-3 cells were infected with WT, Δ678, or Δ3678 virus at an MOI of 0.1 and 1.0, respectively. The images of infected Vero-E6 (**a**) and Calu-3 cells (**b**) were taken at 24 and 48 h post-infection, respectively. Scale bar, 100 μm. The experiments were repeated 3 times with similar results.

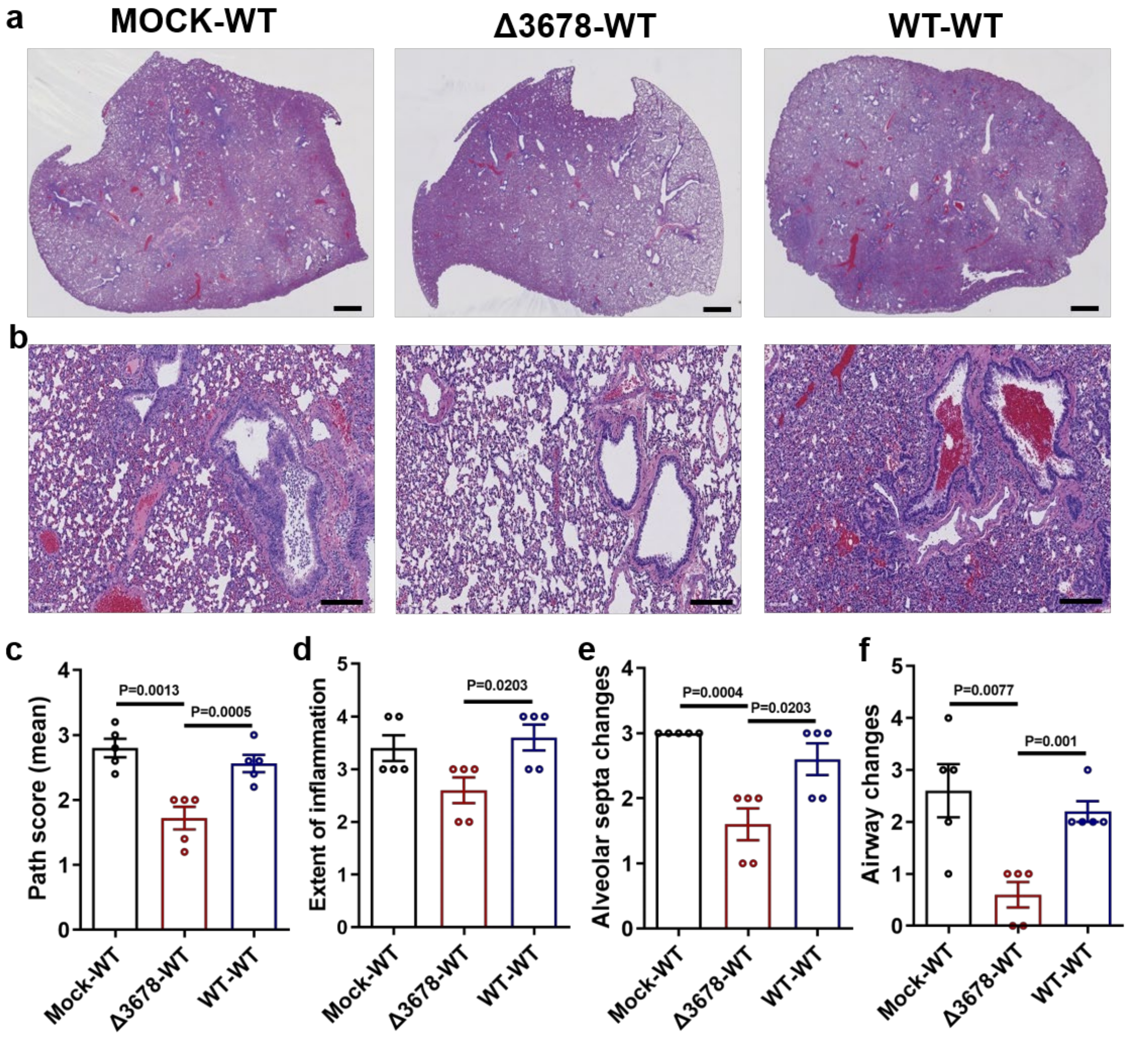


**Supplementary Fig. 2. Lung pathology of Δ3678 virus-immunized and WT SARS-CoV-2-challenged hamsters.**

**a,** Lung sections show typical interstitial pneumonia with moderate to severe inflammatory changes in mock-immunized and WT virus-challenged animals (mock-WT) or in WT virus-inoculated and WT virus-challenged animals (WT-WT). Reduced inflammatory, alveolar septa, and airway changes are observed in Δ3678 virus-immunized and WT virus-challenged animals (Δ3678-WT). Scar bar, 2 mm. **b,** Higher magnification images show large inflammatory cells in the airways and prominent septal thickening in the mock-WT and WT-WT groups. Such changes are minimal or absent in the Δ3678-WT group. Scar bar, 0.5 mm. **c-f,** Comparative pathology scores calculated based on the criteria described in Extended Data Table 2. The Δ3678-WT group shows a significant reduction in total pathology score (**c**), extent of inflammation (**d**), alveolar septa changes (**e**), and airway changes (**f**). Dots represent individual animals (n=5). The values of mean ± standard error of mean are presented. An unpaired two-tailed *t* test was used to determine significant differences between Δ3678 and MOCR or WT groups. *P* values were adjusted using the Bonferroni correction to account for multiple comparisons. Differences were considered significant if $p<0.025$. **c-f** Source Data are provided as a Source Data file.

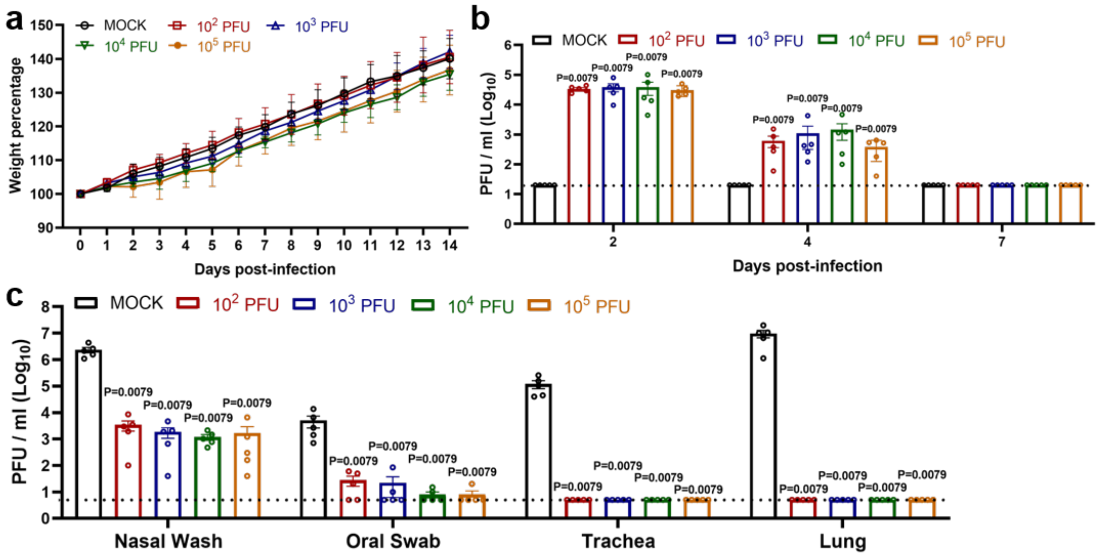


**Supplementary Fig. 3. Dose range immunization of Δ3678 virus to protect hamsters from WT SARS-CoV-2 challenge.**

**a,** Weight loss of hamsters immunized with four different doses of Δ3678 virus. Hamsters were intranasally inoculated with $10^2$, $10^3$, $10^4$, or $10^5$ PFU of Δ3678 virus (n=5 per dose). Body weights were measured for 14 days post-inoculation. The data are shown as mean ± standard deviation. The weight changes were statistically analyzed using two-factor analysis of variance (ANOVA, two-sided) with Tukey's post hoc test. No statistic difference was observed among mock and all Δ3678 dose groups. **b,** Nasal viral loads in Δ3678 virus-immunized hamsters on days 2, 4, and 7 post-immunization. **c,** Viral loads in nasal wash, oral swab, trachea, and lung from Δ3678-immunized and WT virus-challenged hamsters. The Δ3678-immunized hamsters were challenged with WT SARS-CoV-2 on day 28 post-immunization. The viral loads were measured on day 2 post-challenge. **b,c,** Dots represent individual animals (n=5). The values in the graph represent the mean ± standard error of mean. Dash lines indicate assay detection limitations. A non-parametric two-sided Mann-Whitney test was used to determine the statistical differences between mock- and Δ3678-immunized hamsters. *P* values were adjusted using the Bonferroni correction to account for multiple comparisons. Differences were considered significant if p<0.01. **a-c,** Source Data are provided as a Source Data file.

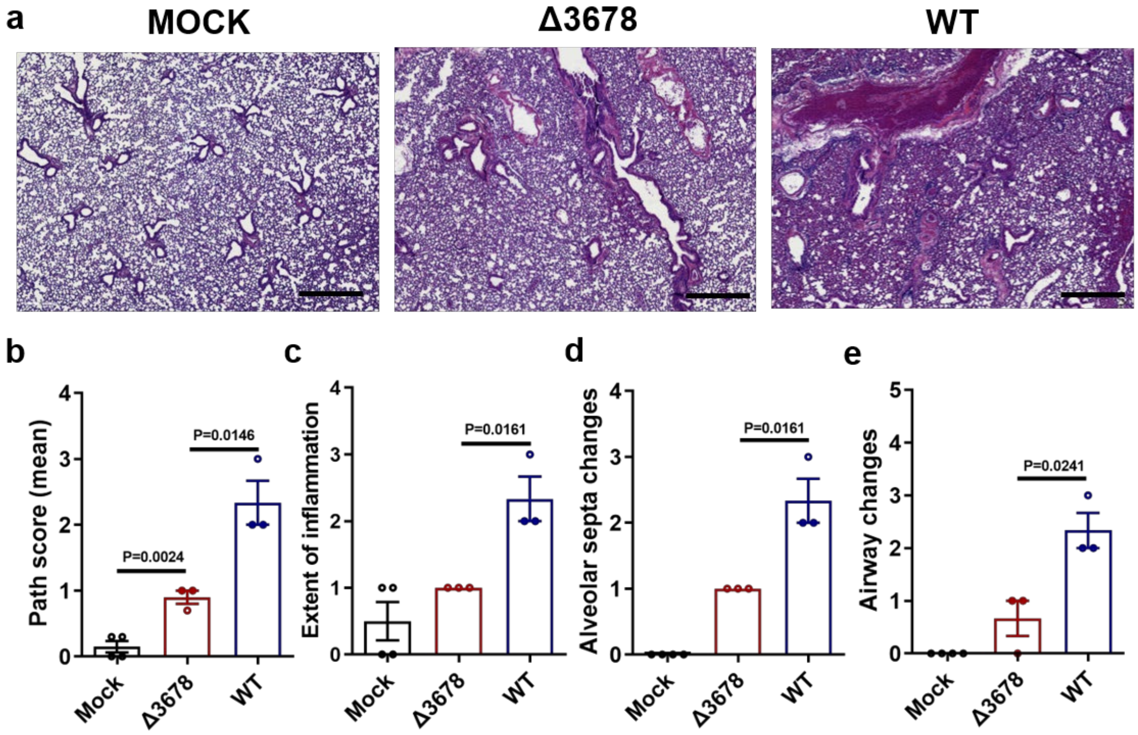


**Supplementary Fig. 4. Lung histopathology of Δ3678 and WT SARS-CoV-2 infected K18-hACE2 mice.**

**a,** WT virus infected lung section shows moderate to severe inflammatory changes, typical of viral pneumonia at day 2 post-infection. The inflammatory, alveolar septa, and airway changes are attenuated in mice infected with Δ3678 virus. The uninfected (Mock) mice served as control. Scar bar, 0.5 mm. **b-e,** Comparative pathology scores calculated based on the criteria described in Extended Data Table 2. The Δ3678 group shows a significant reduction in total pathology score (**b**), extent of inflammation (**c**), alveolar septa changes (**d**), and airway changes (**e**). Dots represent individual animals (n=4 for Mock, n=3 for Δ3678 and WT group). The values of mean ± standard error of mean are presented. An unpaired two-tailed *t* test was used to determine significant differences between Δ3678 and MOCR or WT groups. *P* values were adjusted using the Bonferroni correction to account for multiple comparisons. * Differences were considered significant if p<0.025. **b-e,** Source Data are provided as a Source Data file.

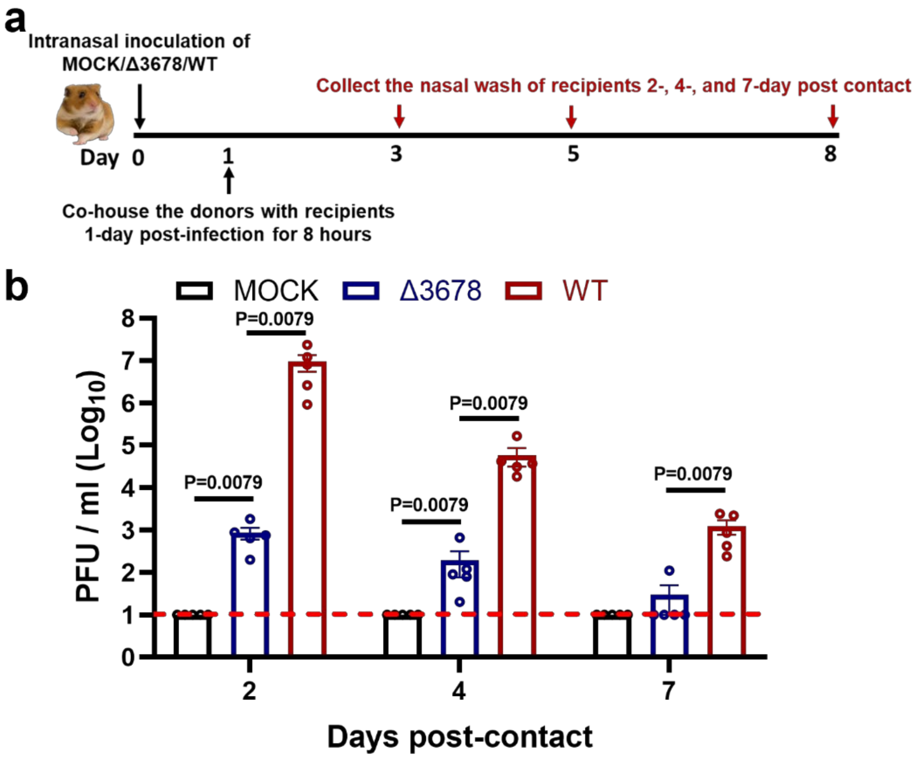


**Supplementary Fig. 5. The transmission of Δ3678 virus-infected hamsters.**

**a,** Experimental design of Δ3678 virus transmission in hamsters. Hamsters were infected with $10^6$ PFU of Δ3678 virus, WT virus or medium mock (n=5 per group). On day 1 post-infection, the donor hamsters were co-housed with clean recipient hamsters for 8 h. The nasal washes of recipient hamsters were collected on days 2, 4, and 7 post-contact. **b,** Viral loads in the nasal washes of recipient hamsters post-contact. Dots represent individual animals. The values in the graph represent the mean ± standard error of mean. A non-parametric two-tailed Mann-Whitney test was used to determine the statistical differences between Δ3678-infected and mock or WT-infected groups. *P* values were adjusted using the Bonferroni correction to account for multiple comparisons. Differences were considered significant if $p<0.025$. Source Data are provided as a Source Data file.

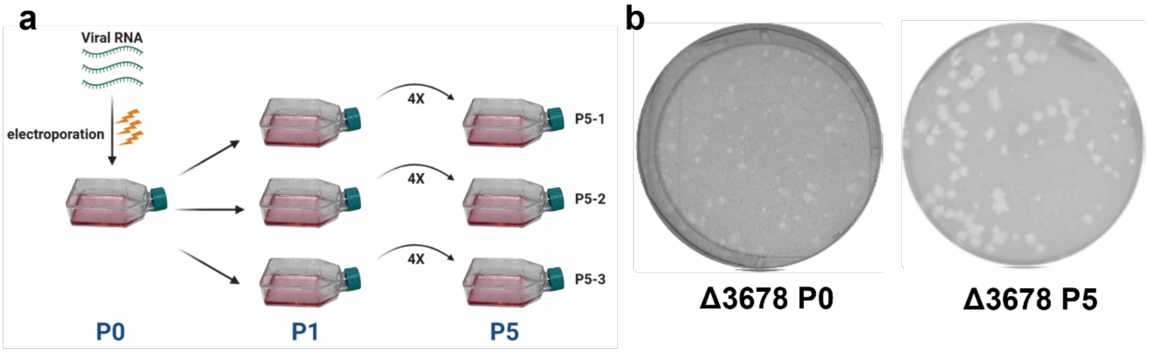


c

| Virus (5 passages) | Replicates | Mutations |
|---|---|---|
| Δ3678 | 1 | Spike H655Y<br>Spike 675-679 QTQTN deletion |
| | 2 | Spike H655Y<br>Spike 675-679 QTQTN deletion |
| | 3 | ORF1ab K2442R<br>ORF1ab I3737M<br>ORF1ab I3738V<br>Spike H655Y<br>Spike 675-679 QTQTN deletion |

**Supplementary Fig. 6. Genetic stability analysis of Δ3678 SARS-CoV-2.**
**a,** Experimental scheme. Passage 0 (P0) Δ3678 virus was divided into three T25 flasks for 5 rounds of independent passaging on Vero-E6 cells. **b,** Plaque morphologies of P0 and P5 Δ3678 virus. **c,** Mutations recovered from three independently cultured P5 Δ3678 viruses. The P5 Δ3678 viral RNAs were extracted and amplified by RT-PCR. Whole-genome sequencing was performed on the RT-PCR products. Mutations from the P5 viruses were annotated with amino acid changes and specific genes. In contrast to the Vero-E6 cell results, when the Δ3678 virus was passaged on Vero-E6-TMPRSS2 cells for 10 rounds, no spike mutations were detected in the P10 virus by NGS analysis; in addition, all the engineered TRS mutations were retained in the P10 virus.

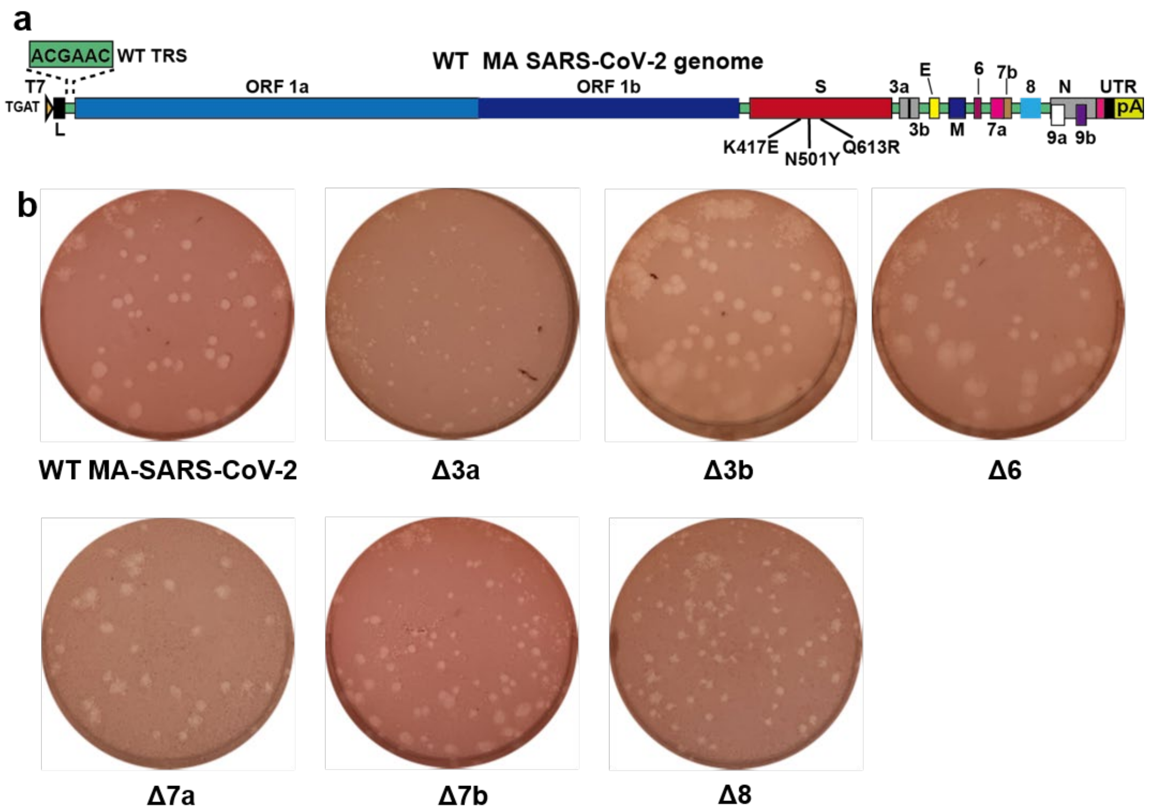


**Supplementary Fig. 7. Construction of mouse-adapted SARS-CoV-2s with individual ORF deletions.**

**a,** An infectious cDNA clone of mouse-adapted SARS-CoV-2 genome. Mouse-adapted SARS-CoV-2 (MA-SARS-CoV-2) contains three amino acid mutations in spike glycoprotein: K417E, N501Y, and Q613R. These mutations confer SARS-CoV-2 to replicate in BALB/c mice. Wild-type TRS sequence is depicted in green. T7, T7 promoter; L, leader sequence; TRS, transcription regulatory sequences; ORF, open reading frame; E, envelope glycoprotein gene; M, membrane glycoprotein gene; N, nucleocapsid gene; UTR, untranslated region; pA, poly A tails. **b,** Plaque morphologies of MA-SARS-CoV-2s with individual ORF deletions. All these ORF deletion viruses were constructed in the backbone of MA-SARS-CoV-2. Plaque assays were performed on Vero-E6 cells and stained on day 2.5 post-infection.

**Supplementary Table 1. Primers used for quantitative real-time RT-PCR.**

| Name | Sequence |
|---|---|
| CoV19-N2-F | TTACAAACATTGGCCGCAAA |
| CoV19-N2-R | GCGCGACATTCCGAAGAA |
| IFN-α-F | TTTCTCCTGCCTGAAGGACAG |
| IFN-α-R | GCTCATGATTTCTGCTCTGACA |
| IFITM1-F | TCATCCTGTCACTGGTATTCGGCTC |
| IFITM1-R | GTGGGTATAAACTGCTGTATCTAGGG |
| ISG56-F | GCTTTCAAATCCCTTCCGCTAT |
| ISG56-R | ACTTCAAGCACCTTTTCAAAGC |
| OAS1-F | CCAAGCTCAAGAGCCTCATC |
| OAS1-R | GAGCTCCAGGGCATACTGAG |
| PKR-F | AAAGCGAACAAGGAGTAAG |
| PKR-R | GATGATGCCATCCCGTAG |
| GAPDH-F | AGGTCGGTGTGAACGGATTTG |
| GAPDH-R | TGTAGACCATGTAGTTGAGGTCA |

**Supplementary Table 2. Criteria for histopathology scoring.**

| Location \ Scores | | 0 | 1 | 2 | 3 | 4 |
|---|---|---|---|---|---|---|
| **A** | Extent of inflammation (% tissue involved) | 0 | <10 | 10-30 | 30-60 | >60 |
| **B** | Inflammatory foci type | No inflammation | Patchy inflammatory foci, few (<2) | Patchy inflammatory foci, many (>2) | Large inflammatory foci, few (<2) | Large inflammatory foci, many (>2) |
| **C** | Alveolar septa | Thin and delicate | Thickened in <10% HPF | Thickened in <30% HPF | Thickened in <60% HPF | Thickened in >60% HPF |
| **D** | Airways | Clear; no cells | Few cells in airway | Moderate cells in airway | More cells in air way; Epithelial hyperplasia | Occlusion of air way/epithelial hyperplasia or desquamation |
| **E** | Alveoli/ perivascular cuff/blood vessels/ pleuritis/cell types | Clear; no inflammatory cells | Few cells. Few PMN or MNC | Moderate cells/ PVC/mild congestion/ mild pleuritis/mostly MNC | More cells/PVC/ more congestion and pleuritis/more MNC and PMN | Abundant cells/large PVC/severe congestion or pleuritis/mixed cells |

HPF – high power field (>10x); PMN – polymorphonuclear cells/heterophils; MNC – mononuclear cells including lymphocytes and macrophages; PVC – perivascular cuff.
