# Supplementary material for: A live-attenuated SARS-CoV-2 vaccine candidate with accessory protein deletions
Source: Nat Commun. 2022 Jul 27;13:4337. doi: 10.1038/s41467-022-31930-z (PMC9326133; doi:10.1038/s41467-022-31930-z)
Supplement: Supplementary file 2 — Reporting Summary [file 41467_2022_31930_MOESM2_ESM.pdf]

nature portfolio

Corresponding author(s): Pei-Yong Shi

Last updated by author(s): Jun 16, 2022

Reporting Summary

Nature Portfolio wishes to improve the reproducibility of the work that we publish. This form provides structure for consistency and transparency in reporting. For further information on Nature Portfolio policies, see our Editorial Policies and the Editorial Policy Checklist.

Statistics

For all statistical analyses, confirm that the following items are present in the figure legend, table legend, main text, or Methods section.

n/a	Confirmed	
☐	☒	The exact sample size (n) for each experimental group/condition, given as a discrete number and unit of measurement
☐	☒	A statement on whether measurements were taken from distinct samples or whether the same sample was measured repeatedly
☐	☒	The statistical test(s) used AND whether they are one- or two-sided<br>*Only common tests should be described solely by name; describe more complex techniques in the Methods section.*
☒	☐	A description of all covariates tested
☐	☒	A description of any assumptions or corrections, such as tests of normality and adjustment for multiple comparisons
☐	☒	A full description of the statistical parameters including central tendency (e.g. means) or other basic estimates (e.g. regression coefficient) AND variation (e.g. standard deviation) or associated estimates of uncertainty (e.g. confidence intervals)
☐	☒	For null hypothesis testing, the test statistic (e.g. F, t, r) with confidence intervals, effect sizes, degrees of freedom and P value noted<br>*Give P values as exact values whenever suitable.*
☒	☐	For Bayesian analysis, information on the choice of priors and Markov chain Monte Carlo settings
☒	☐	For hierarchical and complex designs, identification of the appropriate level for tests and full reporting of outcomes
☐	☒	Estimates of effect sizes (e.g. Cohen's d, Pearson's r), indicating how they were calculated

Our web collection on statistics for biologists contains articles on many of the points above.

Software and code

Policy information about availability of computer code

Data collection	No code and software used for the data collection
Data analysis	Graphpad Prism 9 and ZEN 3.5 (blue edition)

For manuscripts utilizing custom algorithms or software that are central to the research but not yet described in published literature, software must be made available to editors and reviewers. We strongly encourage code deposition in a community repository (e.g. GitHub). See the Nature Portfolio guidelines for submitting code & software for further information.

Data

Policy information about availability of data

All manuscripts must include a data availability statement. This statement should provide the following information, where applicable:

- Accession codes, unique identifiers, or web links for publicly available datasets
- A description of any restrictions on data availability
- For clinical datasets or third party data, please ensure that the statement adheres to our policy

Source data for generating the main figures and supplementary figures are provided with this paper. The mNG reporter Δ3678 SARS-CoV-2 will be deposited to the World Reference Center for Emerging Viruses and Arboviruses (https://www.utmb.edu/wrceva) at UTMB for distribution. Any other information is available upon request.

Field-specific reporting

Please select the one below that is the best fit for your research. If you are not sure, read the appropriate sections before making your selection.

☒ Life sciences ☐ Behavioural & social sciences ☐ Ecological, evolutionary & environmental sciences

For a reference copy of the document with all sections, see nature.com/documents/nr-reporting-summary-flat.pdf

Life sciences study design

All studies must disclose on these points even when the disclosure is negative.

Sample size	No statistical methods were used to pre-determine the sample size. Sample size was chosen based on previous experience and standards in the field.
Data exclusions	No data was excluded in the study.
Replication	The animal experiments were performed once using 5-20 animals and were confirmed using different methods. All attempts at replication were successful.
Randomization	Animals were received by dedicated animal research personnel at UTMB, who randomly assigned the animals to cages with no additional knowledge of study design. No further randomization was performed by research personnel.
Blinding	The investigators were not blinded to the allocation during the experiments or to the outcome assessment. Blinding is not necessary because the results are quantitative and did not require subjective judgment or interpretation. Blinding is not typically used in the field.

Reporting for specific materials, systems and methods

We require information from authors about some types of materials, experimental systems and methods used in many studies. Here, indicate whether each material, system or method listed is relevant to your study. If you are not sure if a list item applies to your research, read the appropriate section before selecting a response.

Materials & experimental systems

n/a	Involved in the study	
☐	☒	Antibodies
☐	☒	Eukaryotic cell lines
☒	☐	Palaeontology and archaeology
☐	☒	Animals and other organisms
☒	☐	Human research participants
☒	☐	Clinical data
☒	☐	Dual use research of concern

Methods

n/a	Involved in the study	
☒	☐	ChIP-seq
☒	☐	Flow cytometry
☒	☐	MRI-based neuroimaging

Antibodies

Antibodies used	1. Anti-STAT1 (cellsignal, 14994S, 1:1,000),<br>2. Anti-pSTAT1 (Y701) (cellsignal, 7649S, 1:1,000),<br>3.Anti-STAT2 (cellsignal, 72604S, 1:1,000),<br>4.Anti-pSTAT2 (Y690) (cellsignal, 88410S, 1:1,000),<br>5.Anti-GAPDH (sigmaaldrich, G9545, 1:1,000),<br>6.SARS-CoV-2 (COVID-19) nucleocapsid antibody (novusbio, NB100-56576, 1:1000),<br>7. IgG14 (CoV2-14) was isolated from phage-displayed antibody library(Ku, Z., et al. 2021).
Validation	1.https://www.cellsignal.com/products/primary-antibodies/stat1-d1k9y-rabbit-mab/14994?_=1651711054957&Ntt=(14994S&tahead=true<br>2.https://www.cellsignal.com/products/primary-antibodies/phospho-stat1-tyr701-d4a7-rabbit-mab/7649?site-search-type=Products&N=4294956287&Ntt=7649s&fromPage=plp&_requestid=8578217<br>3. https://www.cellsignal.com/products/primary-antibodies/stat2-d9j7l-rabbit-mab/72604?site-search-type=Products&N=4294956287&Ntt=72604s&fromPage=plp&_requestid=8578261<br>4. https://www.cellsignal.com/products/primary-antibodies/phospho-stat2-tyr690-d3p2p-rabbit-mab/88410?site-search-type=Products&N=4294956287&Ntt=88410s&fromPage=plp&_requestid=8578302<br>5. https://www.sigmaaldrich.com/US/en/product/sigma/g9545<br>6. https://www.novusbio.com/products/sars-nucleocapsid-protein-antibody_nb100-56576<br>7. IgG14 (CoV2-14) was validated by Ku, Z., et al. 2021.

Eukaryotic cell lines

Policy information about cell lines

Cell line source(s)	Vero E6 cells (CRL-1586) and Calu-3 cells (HTB-55) were obtained from ATCC; Vero-E6-TMPRSS2 cells were purchased from SEKISUI XenoTech, LLC. The HAE cells were purchased from MatTek Life Science. The A549-hACE2 cells that stably express hACE2 were generously provided by Shinji Makino.
Authentication	ATCC have comprehensively performed authentication on cell lines through STR profiling. Vero-E6-TMPRSS2 cells have been authenticated by SEKISUI XenoTech; the HAE cells have been authenticated by MatTek life Science. The A549-hACE2 cells were not authenticated.
Mycoplasma contamination	All cell lines were tested negative for mycoplasma.
Commonly misidentified lines (See ICLAC register)	No commonly misidentified cell lines were used in the study.

Animals and other organisms

Policy information about studies involving animals; ARRIVE guidelines recommended for reporting animal research

Laboratory animals	Four- to six-week-old male golden Syrian hamsters, strain HsdHan:AURA (Envigo, Indianapolis, IN), and 8- to 10-week-old K18-hACE2 female mice from Jackson Laboratory (Bar Harbor, ME) and 8- to 10-week-old BALB/c female mice from Charles River Laboratories (Wilmington, MA) were used for animal study.
Wild animals	No wild animals were used in this study.
Field-collected samples	No filed-collected samples were used in this study.
Ethics oversight	Hamster and mouse studies were performed in accordance with the guidance for the Care and Use of Laboratory Animals of the University of Texas Medical Branch (UTMB). The protocol was approved by the Institutional Animal Care and Use Committee (IACUC) at UTMB. All the animal operations were performed under anesthesia by isoflurane to minimize animal suffering.

Note that full information on the approval of the study protocol must also be provided in the manuscript.
